# Aquatic Bacterial Community Responses to Aquatic Contaminants Revealed by 16S rRNA Metabarcoding in Field‐Based Microcosms

**DOI:** 10.1111/1758-2229.70328

**Published:** 2026-03-27

**Authors:** A. S. Flynn, A. M. Osborn, V. Pettigrove, J. Shimeta, S. M. Long

**Affiliations:** ^1^ Aquatic Environmental Stress Research Group RMIT University Bundoora Victoria Australia; ^2^ School of Science RMIT University Melbourne Victoria Australia

**Keywords:** bioindicators, biomonitoring, environmental DNA, metals, pesticides, pharmaceuticals, sediment, wetlands

## Abstract

In this study we used environmental DNA metabarcoding and field‐based microcosms to assess three classes of aquatic contaminants (metals (copper), pesticides (diuron), and pharmaceuticals (venlafaxine)) and their impact on the structure of freshwater wetland sedimentary bacterial communities. Our results showed that copper had the most influence on bacterial community structure, followed by venlafaxine, then diuron. We also saw that the addition of copper facilitated the release of other sediment‐bound metals (barium, zinc, and vanadium), also having significant impacts on community structure. The bacterial communities were mostly dominated by Proteobacteria, Cyanobacteria, Bacteroidota, and Verrucomicrobiota, which were key drivers of variation among treatments, along with Actinobacteriota. Our findings indicate that the ideal taxonomic level for the assessment and identification of bacterial bioindicators is family, with constraints at lower taxonomic levels. We identified five phyla, 13 families, and three species which show strong potential to be either diagnostic bioindicators of one or more of the chemicals assessed or broad bioindicators of common urban contaminants, eight of which are novel bioindicators. Our study highlights the effectiveness of eDNA metabarcoding to efficiently characterise sedimentary bacterial community assemblages and emphasises its value in aquatic ecosystem assessments, particularly for the prediction of contaminants driving ecosystem change.

## Graphical abstract

This study used sedimentary eDNA analysis and field‐based microcosms to assess the impacts of three major aquatic contaminants on bacterial assemblage structure. Our results showed significant but varied impact depending on exposure to which contaminant; we suggest five phyla, 13 families, and three species as potential bacterial bioindicators and discuss the ideal taxonomic level for bacterial bioindicator identification and assessments. This work emphasises the value of eDNA analysis in aquatic ecosystem assessments and biomonitoring.

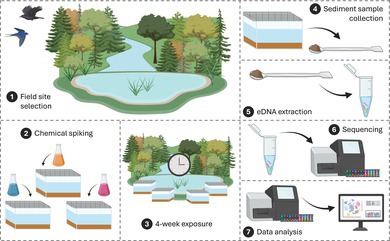

## 1 Introduction

Understanding the impact of common aquatic pollutants on the health and functioning of freshwater ecosystems is increasingly important as global contamination levels continue to rise and freshwater ecosystems are progressively degraded (Amoatey and Baawain 2019; Gál et al. 2019). Wetland ecosystems, both natural and constructed, provide important ecological and biogeochemical services across urban and rural environments (Ilyas and Masih 2017; Metcalfe et al. 2017). The sediments within wetlands often function as sinks for pollutants that enter as a result of surface runoff (Pawlowski et al. 2022). These sediments are sites of important microbially‐driven biogeochemical and metal cycling and where biodegradation of some organic pollutants also occurs (Kivaisi 2001). The toxicity of sedimentary contaminants to microbial assemblages is influenced by the bioavailability of the contaminant, chemical properties of contaminant mixtures, geochemical characteristics of the sediment, interaction mechanisms between the pollutant and affected (micro)‐organisms, and the sensitivity of the affected organisms (Simpson and Batley 2016; Simpson et al. 2005).

There are many legacy contaminants, such as heavy metals, which continue to threaten aquatic ecosystems (Dias‐Ferreira et al. 2016; Guan et al. 2016), as well as the ever‐increasing presence of emerging contaminants, including micropollutants such as pesticides and pharmaceuticals and personal care products (PPCPs); the impacts of which are being increasingly understood (Oluwole et al. 2020; Silva 2016). The sources of heavy metal contamination in freshwater are diverse, originating from mining activities, agricultural practices, industrial effluent, and road runoff, often transported through urban stormwater (Dias‐Ferreira et al. 2016; Guan et al. 2016; Zhang et al. 2016). Increasing detection of pesticides in water bodies could be attributed to their increased use in both agricultural and domestic settings (Delcour et al. 2015) as well as altered atmospheric deposition and altered surface runoff (Kattwinkel et al. 2011). Part of the increase in PPCP detections can be attributed to better detection methods, with the most common sources being from untreated and treated waste water (Gaw et al. 2014; Wilkinson et al. 2022).

The use of environmental DNA (eDNA) analysis as a bioindicator for aquatic pollution is a relatively recent innovation (Apothéloz‐Perret‐Gentil et al. 2017) that has a wide scope of applications (Pawlowski et al. 2021). Analyses of the structure, diversity, and composition of microbial assemblages and their application as bioindicators are becoming more common within aquatic monitoring due to microbes' typically high abundances and high sensitivity and/or response to contaminants (Liao et al. 2020; Liu et al. 2020). Previously, we have used eDNA analysis to investigate variations in bacterial communities across diverse constructed wetlands (comparing communities from wetlands with varying levels of contamination and comparing responses to a copper pollution event between communities originating from clean and historically contaminated environments), showing clear and identifiable shifts in assemblage structure correlated with the presence of chemical pollution mixtures and, in particular, of metal pollution (Flynn et al., n.d.; Flynn et al. 2025).

This current study investigated whether and how microbial (bacterial) community taxonomic signals (at different taxonomic levels) can be detected and how they vary in freshwater wetland sediments in response to exposure to different chemical contaminants at multiple concentrations. 16S rRNA gene eDNA metabarcoding was used to analyse bacterial communities in field‐based wetland sediment microcosms spiked with varying concentrations of either a metal, pesticide, or pharmaceutical pollutant to identify taxonomic signatures responding to pollutant inputs.

## 2 Methods

### 2.1 Experimental Design

Microcosm experiments were established at Glynns wetland (37°44′24.4″S 145°11′43.9″ E), north‐east Melbourne, Australia. The wetland is approximately 200 m long and 20 m wide and was selected due to its location within a state park, rendering it largely unpolluted, with metals present at concentrations below national guideline concentration values for the protection of aquatic species, and pesticides below the limit of detection (Table S1) as its small catchment area sits entirely within natural parkland. The wetland area also has limited public access, reducing the probability of any unwanted interactions with the experiment. This research was conducted under Parks Victoria Access Agreement AA‐0002007 (variation VAR‐00001184).

**FIGURE 1:**
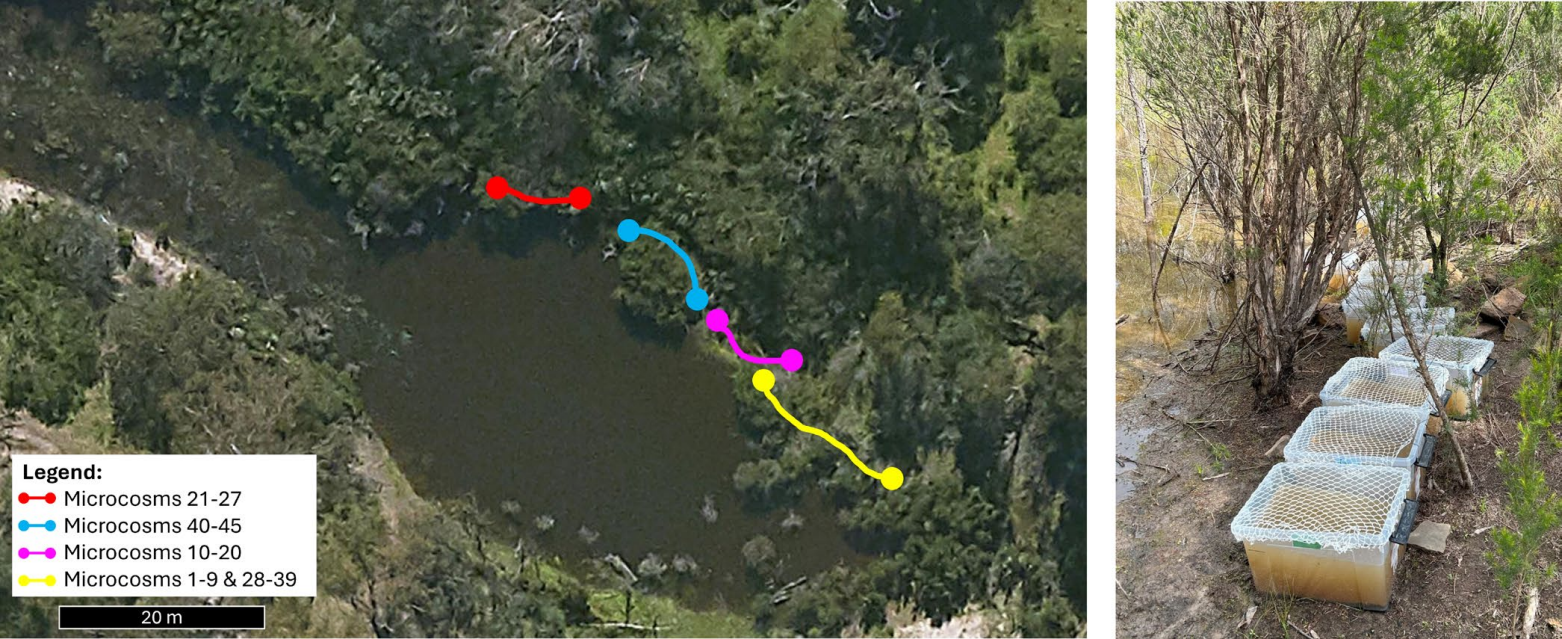
Map of the field site and placement of the microcosms along the wetland bank (left) and microcosm set‐up one week after deployment (right).

A total of 7 treatments across 45 microcosms were used: C (control, no spike); M‐L (metal spiked (copper), low concentration); M‐H (metal spiked (copper), high concentration); Pe‐L (pesticide spiked (diuron), low concentration); Pe‐H (pesticide spiked (diuron), high concentration); Ph‐L (pharmaceutical spiked (venlafaxine), low concentration); Ph‐H (pharmaceutical spiked (venlafaxine), high concentration). Each experimental (pollutant) treatment had 5 replicates except for the control which had 15 replicates. The microcosms were aligned along the bank of the wetland in random order, as shown in Figure 1.

The three chemicals (copper, diuron, and venlafaxine) were selected respectively as representatives of three major groups of aquatic contaminants (metals, pesticides, and pharmaceuticals) occurring globally. These chemicals in particular were chosen due to their recent prevalence across Melbourne wetlands and other surface waters, making them contaminants of concern in Melbourne (Flynn et al. 2025; Pettigrove et al. 2023; Saaristo et al. 2024). From previous research, we expect that copper and diuron will have an effect on microbial community structure (Flynn et al. 2025; Lear et al. 2023; Mahamoud Ahmed et al. 2020; Prado and Airoldi 2001). While there is no current research investigating the effects of venlafaxine on environmental microbes, given that it has been shown to alter gut microbial composition (Rukavishnikov et al. 2023; Shen et al. 2023), we hypothesise that venlafaxine may also directly affect sedimentary microbes.

### 2.2 Microcosm Preparation & Deployment

To prepare the sediments for the microcosms, surficial sediments were collected from Glynns wetland prior to the beginning of the experiment and filtered through a 63 μm net. The sediments were settled for a week at 4°C, with the overlying water decanted after this time. To prepare the microcosm tubs, 20 L plastic tubs were labelled with treatment, assigned a randomly generated number between 1 and 45 (Table S2), and a 15 L fill line mark was made. Three pollutant‐spiking stock solutions were prepared for copper (CuSO_4_), diuron, and venlafaxine (venlafaxine hydrochloride), each at a concentration of 10 mg/L.

The spiking concentrations for the metal treatments were determined from the ANZG (2018) freshwater guideline values, using the 80% trigger value for species protection (2.5 μg/L) for the M‐H treatment and the 95% trigger value (1.4 μg/L) for the M‐L treatment. No freshwater guidelines for diuron are available in Victoria, so instead the spiking concentrations for the pesticide treatments were determined from the Queensland Department of Science, Information Technology and Innovation‐proposed freshwater guideline values, using the 80% trigger value (0.9 μg/L) for the Pe‐H treatment and the 95% trigger value (0.23 μg/L) for the Pe‐L treatment (King et al. 2017). Spiking concentrations for the pharmaceutical treatments were determined from a recent survey of waterways in Victoria, Australia, with the highest detection of venlafaxine in that study (3 μg/L) being used for the Ph‐H treatment and the lowest detection (0.97 μg/L) being used for the Ph‐L treatment (Saaristo et al. 2024). The final spike volumes used were increased from the base values described above to account for up to 95% movement into the sediment (Table S3).

This experiment commenced on 17th October 2024 and was run for four weeks, finishing on 12th November 2024. Sediment microcosms were established in polypropylene tubs with overlying site water, as used previously in Yang et al. (2018). In this current experiment, the set‐up was established by placing the empty microcosm tubs in a single‐file line along the edge of the wetland, as shown in Figure 1, before 500 mL of the filtered sediment was added to each tub. Filtered (63 μm) site water was then added, up to the 15 L fill mark. Once filled, each microcosm was spiked with its corresponding solution and volume (Table S3) and gently stirred to minimise sediment disturbance. Bird netting was placed over each of the microcosms to stop larger debris from falling into the microcosms while allowing for open interactions with the atmosphere and invertebrates (Pettigrove and Hoffmann 2005). The microcosms were checked weekly during deployment where water quality measurements (temperature, pH, turbidity, electrical conductivity, dissolved oxygen) were recorded and were re‐filled to the original water line with filtered site water if necessary.

### 2.3 Sample Collection

To sample a microcosm, first a sediment sample for DNA analysis was collected. This was done using a long‐handled spoon to collect surface sediment from five random areas of the microcosm, at least 2 cm inwards from the edge, with samples aliquoted into labelled 2 mL Eppendorf tubes. Tubes were shaken to homogenise samples and stored in a cooled polystyrene container in the dark, prior to transfer to the laboratory and storage at –80°C.

Surface water samples were then collected directly into 100 mL amber glass bottles for pesticide and pharmaceutical analysis. Samples for metal analysis were collected via a 0.22 μm filter into 60 mL plastic bottles containing nitric acid preservative, samples for nutrient analysis into 60 mL plastic bottles containing sulphuric acid preservative, and samples for major cation analysis into 100 mL plastic bottles with no preservatives. All the sample collection bottles were prepared and provided by the laboratories which handled the analysis. After the water samples were collected, the remaining overlying water was decanted through a 63 μm sieve to collect any macroinvertebrates or debris before disposal. The remaining sediment was then poured through the same sieve into a sediment collection bucket. Each replicate sediment sample was poured into the same collection bucket, creating a pooled sample. Larger debris was removed from the sieve, then any remaining debris or macroinvertebrates were collected and stored in 100% ethanol until processing in the lab. The collected sediment was left to settle at 4°C for 3 days before samples were collected into amber jars for analysis. This was repeated for every microcosm, starting with the controls and then followed by the other treatments.

In addition to the samples collected upon completion of the experiment, sediment samples were collected before deployment of the microcosms (day 0) from the pooled sediment which was to be subsequently portioned for use in the microcosms.

### 2.4 Chemical Analysis, DNA Extraction & Sequencing

Chemical analysis of the sediment and water was completed at Australian Laboratory Services (ALS) (Springvale, Australia) for metals, pesticide, and nutrient/major cations analyses. The metals (chromium, cobalt, copper, lead, manganese, arsenic, nickel, selenium, beryllium, vanadium, zinc, barium, boron, cadmium) were analysed by Inductively Coupled Plasma‐Atomic Emission Spectroscopy (ICP‐AES) and mercury by Cold Vapour‐Flow Injection Mercury System (CV/FIMS), pesticides (diuron) by Liquid Chromatography/Mass Spectrometry–Mass Spectrometry (LC‐MS/MS), and nutrients/major cations (calcium, magnesium, sodium, potassium, hardness (CaCO_3_), total nitrogen, nitrite, ammonia, nitrate, reactive phosphorus, total phosphorus, Total Kjeldahl nitrogen) as described in Jeppe et al. (2017). Pharmaceutical analysis of the sediment and water was done at the National Measurement Institute (NMI) (Port Melbourne, Australia). The pharmaceuticals (venlafaxine) were analysed by Liquid Chromatography/Mass Spectrometry (LC‐MS), as described in Method S1.

DNA was extracted from 250 mg of sediment from each sample with a DNeasy PowerSoil Pro extraction kit (Qiagen, Clayton, Australia), using the spin‐column protocol (Qiagen 2023). Samples were extracted 24 at a time (two batches of 24, one of five) including a negative control (nuclease‐free water) in each. The eluted DNA samples were analysed by a NanoDrop microvolume spectrophotometer (Thermo Fisher, Scoresby, Australia) for purity and quantity, and to ensure no DNA contamination in the controls. The samples were diluted to 5–10 ng DNA/μL and 20 μL pipetted into a 96‐well plate, one sample per well, arranged in columns, and stored at −80°C until analysis.

The samples were sent to the Ramaciotti Centre for Genomics (UNSW, Sydney, Australia) for 16S v3‐v4 amplicon library preparation (Illumina 2013) and a 2x300bp paired‐end llumina MiSeq sequencing run. The library preparation and sequencing analysis were completed according to the manufacturer's protocols. Library preparation was as follows: amplicon PCR (95°C for 3 min; 25 cycles of: 95°C for 30 s, 55°C for 30 s, 72°C for 30 s; 72°C for 5 min; Hold at 4°C) using Illumina overhang primers and locus‐specific primers (16S_341f, 16S_805r); amplicon cleaning using AMPure XP beads (Beckman Coulter, Mount Waverly, Australia); indexing PCR with Nextera compatible IDT10 unique dual indexes (N7XX, S5XX); sample normalisation using SequalPrep kit (Thermo Fisher, Scoresby, Australia); sample elution in 20 uL buffer with equal volumes used for pooling; and pooled sample cleaning using AMPure XP beads (Beckman Coulter, Mount Waverly, Australia). Quantification and molarity calculations were determined by TapeStation (Agilent, Santa Clara, USA) and Qubit (Thermo Fisher, Scoresby, Australia).

### 2.5 Bioinformatics & Microbial Statistical Analysis

Bioinformatic analysis of the genomic sequences was done using the QIIME 2 (v 2022.8) platform (Bai et al. 2019; Bolyen et al. 2019), run through the RMIT University Amazon Web Services (AWS) cloud supercomputing (RACE) hub. First, primers were removed using the q2‐cutadapt plugin (Martin 2011) followed by filtering, trimming, and denoising into amplicon sequencing variants (ASVs), using the trim parameters trim‐left 5 for forward and reverse sequences and trunc‐len 240 for forward and trunc‐len 220 for reverse sequences. Chimeric sequences were then removed, using the DADA2 (v. 1.16.0) pipeline (Callahan et al. 2016), and any samples with < 10,000 reads were removed. Taxonomy was assigned using the Greengenes2 (v 2022.10) classifier (McDonald et al. 2023) and a phylogenetic tree constructed from masked alignments using the q2‐phylogeny plugin. Alpha diversity indices (evenness, Shannon diversity & Faith's PD) were calculated with the q2‐diversity plugin on data rarefied to a sampling depth of 100,000 sequences. The classified ASV relative frequency tables were exported from QIIME and imported into PRIMER‐e (v7) with PERMANOVA+ for multivariate analyses along with the raw chemical data.

In PRIMER (Clarke and Gorley 2015), a Bray‐Curtis resemblance matrix (Bray and Curtis 1957) was constructed from log (x + 1) transformed biological data, while the chemical data was Z‐score normalised. The following analyses were performed on the resemblance matrix and transformed data sets: non‐metric multidimensional scaling (nMDS), PERMANOVA, analysis of similarities (ANOSIM), SIMPER analysis (Clarke 1993), distance‐based redundancy analysis (dbRDA), and BEST analysis (Clarke et al. 2014). A significance level of *α* = 0.05 was used in all analyses. Where available, chemicals were compared to Australian and New Zealand freshwater toxicity guideline values (ANZG 2018) or the Queensland Department of Science, Information Technology and Innovation proposed guidelines (King et al. 2017).

### 2.6 Macroinvertebrate Identification & Statistical Analysis

Aquatic macroinvertebrates were identified to the lowest possible taxonomic level under a dissecting microscope using the Centre for Freshwater Ecosystems bug identification key (Hawking et al. 2013). Analyses were performed in PRIMER‐e (v7) and Minitab (v21.4.3). Only taxa which were present in at least 10% of replicates were included in statistical analysis. An nMDS ordination plot was used to visualise the variation between treatments, with a *t*‐test used to determine differences in mean abundances. Spearman's rank correlation and a Kruskal–Wallis test with post hoc Dunn's test were used to assess variation between treatments for each taxon individually.

## 3 Results

### 3.1 Contaminant Concentrations Between Treatments

**FIGURE 2:**
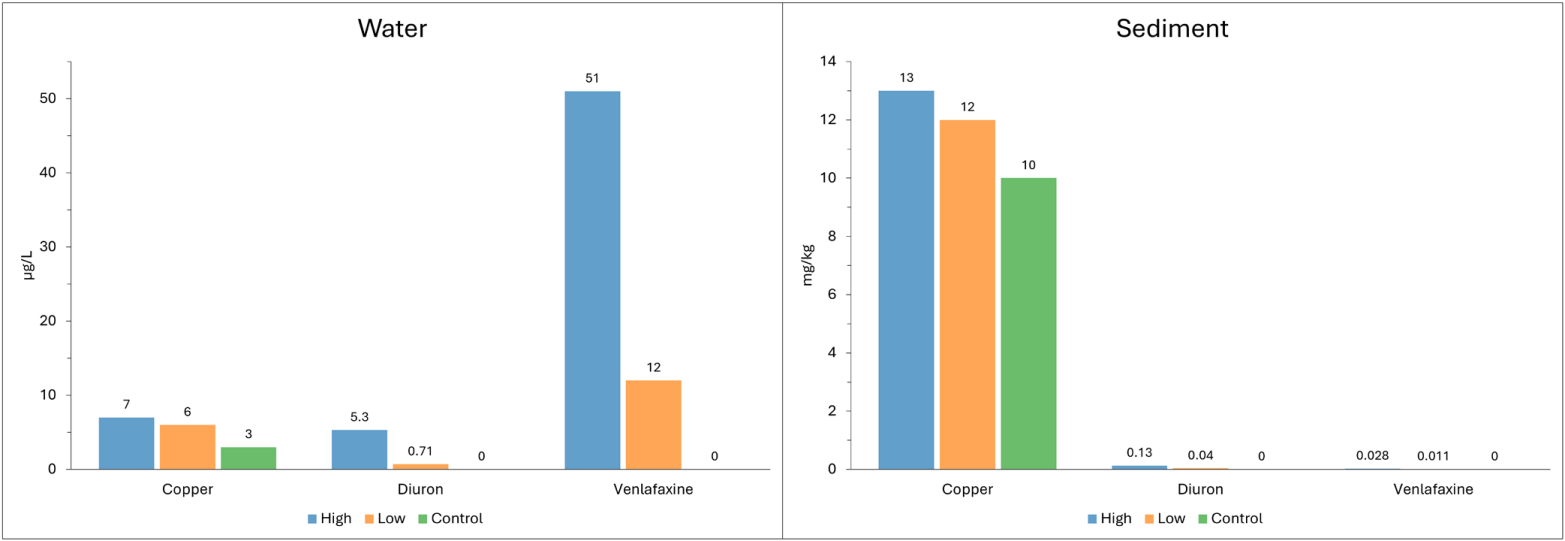
Concentrations in microcosms of copper, diuron, and venlafaxine measured from water (left) and sediment (right) for high and low concentration treatments and in controls. A red asterisk (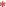) indicates a concentration that exceeds its guideline value (ANZG 2018; King et al. 2017). No guidelines are available for diuron (sediment) or venlafaxine. Note different units of measurement on y axes between plots.

Copper concentrations measured at the end of the experiment in the control and in both the metal high and metal low spiked treatments were below the default guideline value (65 mg/kg) in the sediment but above the 80% trigger value (2.5 μg/L) in the water (Figure 2, Table S4). Diuron and venlafaxine concentrations were below the limit of detection (LoD) in both the sediment and water control samples (Figure 2). Diuron concentrations in the pesticide‐high treatment were above the proposed 80% trigger value (0.9 μg/L) in the water, but below the guideline in the pesticide‐low treatment (Figure 2, Table S4). Sediment diuron concentrations were < 0.15 mg/kg for both spiked treatments (Figure 2). Venlafaxine concentrations in the water were the highest measured of the spiked chemicals, also with the greatest difference between the high and low treatment concentrations (Figure 2). Sediment venlafaxine concentrations were < 0.03 mg/kg for both spiked treatments (Figure 2). Currently, there are no sediment guideline values for diuron or venlafaxine, and no water guideline value for venlafaxine in Australia. There was less movement of the chemicals from the water into the sediment than was estimated from their respective soil mobilities; however, each spiked chemical had the expected relative concentrations of highest concentration in the high sample, mid‐level concentration in the low sample, and lowest concentration in the control sample.

Of the remaining 14 metals measured (other than copper), four metals (mercury, cadmium, beryllium, selenium) were not detected in either the water or sediment in any sample. Arsenic and boron were each only detected in the water low sample, then not detected in any other sample (Table S5a,b). Chromium was present at 1 μg/L in all water samples (Table S5a) and also detected in all sediment samples (Table S5a). Cobalt was below the Limit of Detection (LoD) in all of the water samples (Table S5a) and present at 6 mg/kg in all sediment samples (Table S5b). Nickel, lead, and vanadium were below the LoD in all water samples (Table S5a) but detected in all sediment samples (Table S5b). Manganese, zinc, and barium were detected in all water and sediment samples, with barium having the highest concentrations detected of all metals in the water samples (Table S5a) and zinc and barium having equal highest concentrations detected of all metals in the sediment samples (Table S5b). Of the metals detected in the sediment, all were below their ANZG (2018) guideline values or had no guideline value (Table S5b). Of the metals detected in the water, chromium was present at a concentration equal to the ANZG (2018) 95% trigger value in all treatments and zinc was above the 95% trigger value in two treatments, while the remaining samples had concentrations either below guideline values or there was no guideline value available (Table S5a). Overall, metal concentrations were slightly higher in the copper spiked samples compared to the control sample and were higher in the water samples compared to the sediment samples.

Water quality measurement results are presented in Table S16. No strong temporal effects or variation between treatments were observed over the duration of the experiment.

### 3.2 High‐Throughput Sequencing (HTS) Overview

Sequencing of 16S rRNA genes generated a total of 15,337,248 raw paired‐end reads from DNA extracted from across 50 sediment samples. Following quality control, denoising and removal of potential chimeric sequences, 7,608,633 sequences remained. The average number of eligible reads per sample was 161,885 (min: 107,465, max: 274,448). From these high‐quality reads, 85 phyla, 225 classes, 580 orders, 1086 families, 2127 genera and 2895 species were identified.

### 3.3 Microbial Assemblage Diversity

**FIGURE 3:**
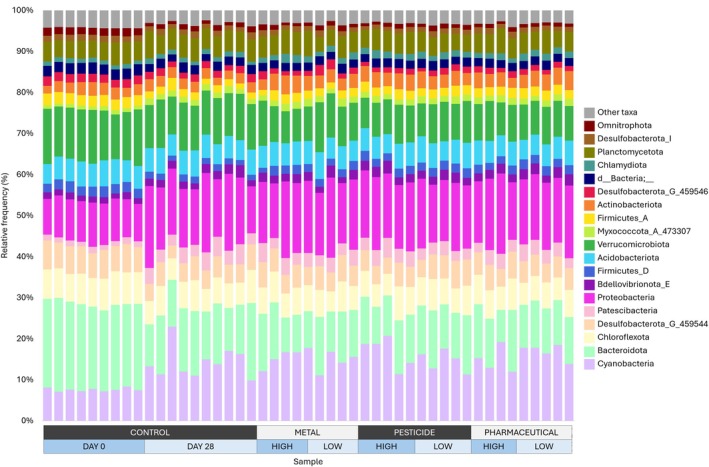
Relative frequency bar plot of all samples shown at phylum level based on 16S rRNA gene sequencing. All samples except Day 0 samples were sampled at the end of the 28‐day experiment. Only significantly abundant taxa (> 1% overall abundance) are displayed separately; all other taxa are combined into ‘other taxa’.

The majority of the high‐quality reads were classified as Bacteria (7,596,459; 99.84%), with the remaining being classified as Archaea (6847; 0.09%) or unassigned (5327; 0.07%). Of the 85 phyla, 16 were considered significantly abundant (> 1% of total abundance). Proteobacteria was the most dominant with 14.9% of total relative abundance, followed by Cyanobacteria and Bacteroidota with 13.9% and 13.8% of total relative abundance, respectively. Verrucomicrobiota accounted for 10.2%, Desulfobacterota for 8.8%, and Chloroflexota for 6.6% of total relative abundance. Acidobacteriota, Planctomycetota, Firmicutes, Actinobacteriota, Patescibacteria, an unassigned bacterium, and Bdellovibrionota each contributed to between 2% and 6% of total relative abundance, while Myxococcota, Chlamydiota, and Omnitrophota each contributed to 1% and 2% of total relative abundance. The relative abundance of each phylum was largely stable across all samples. Proteobacteria, Cyanobacteria, and Patescibacteria showed a slight increase from the day 0 control samples when compared to all the other samples, with Bacteroidota showing the reverse trend (Figure 3).

### 3.4 Variation of Sediment Bacterial Communities Between Control and Pollutant Treatments

Comparing alpha diversity metrics between the bacterial communities within controls and each treatment found no significant differences in evenness, Shannon diversity, or biodiversity (Faith's PD) (Table S6).

**FIGURE 4:**
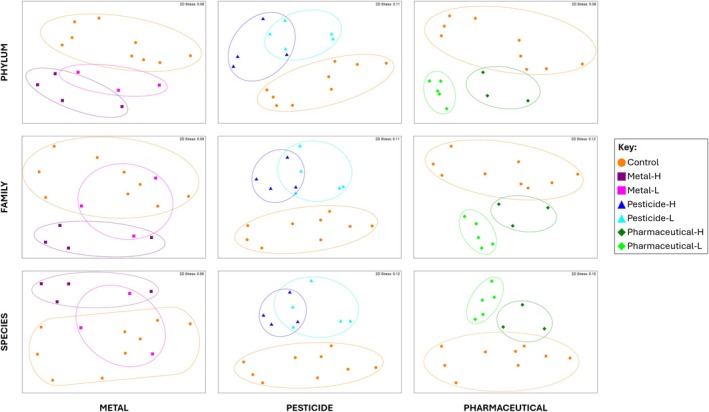
nMDS plot showing variation in the bacterial communities in the metal, pesticide, and pharmaceutical treatments compared to the controls at the phylum, family, and species levels. ANOSIM results are represented by the coloured circles, where no overlap indicates a significant difference between those groups.

The nMDS ordination plots show a clear separation between the control samples and spiked samples across the pesticide and pharmaceutical treatments at all three taxonomic levels investigated (Figure 4). The control and metal treatments were more separated at phylum level, but there was a slight overlap of these communities at family and species level. At all taxonomic levels, the metal and pesticide samples clustered into concentration treatments, although the clusters overlapped somewhat. Conversely, at all taxonomic levels, the pharmaceutical samples clustered into very distinct concentration treatments which did not overlap.

PERMANOVA analysis showed that there were significant differences (*p* ≤ 0.05) between communities with respect to chemical types and between concentration treatments at all taxonomic levels (Table 1). Comparisons of the pairwise chemical and concentration groups reveal that at phylum level the control communities were significantly different from those present in all spiked treatments (Table S7a). At family and species level the control communities were significantly different from those in all spiked treatments except for metals, for which the difference was nearly significant (Table S7a). Across all taxonomic levels no spiked treatments were significantly different from another treatment (Table S7a). Of the three chemicals used, only the Ph treatments were significantly different between its two concentrations at all taxonomic levels, although the Pe treatment was near significance at phylum level (Table S7b). Repeating this assessment with ASV data returned similar results, with the control groups being significantly different from the pesticide and pharmaceutical treatments (Table S8a), and although no concentration pair was significant, they showed the same trend of *p*‐values as at taxonomic levels (Table S8b).

**TABLE 1:** PERMANOVA results comparing variation between bacterial communities between treatment and concentration groups for phylum, family, and species.

	df	SS	MS	Pseudo‐F	P (perm)	Unique perms
**Phylum**						
Treatment	3	118.48	39.494	2.6614	0.005**	997
Concentration (Treatment)	3	104.12	34.706	2.3388	0.008**	999
Res	26	385.82	14.839			
Total	32	613.45				
**Family**						
Treatment	3	427.15	142.38	1.7871	0.008**	999
Concentration (Treatment)	3	340.16	113.39	1.4232	0.044*	998
Res	26	2071.5	79.672			
Total	32	2856				
**Species**						
Treatment	3	745.14	248.38	1.6187	0.004**	995
Concentration (Treatment)	3	615.68	205.23	1.3375	0.04*	998
Res	26	3989.6	153.45			
Total	32	5376				

Abbreviations: df, degrees of freedom; MS, mean sum of squares; P(perm), *p*‐values based on max 999 permutations (lowest possible *p*‐value = 0.001); Pseudo‐F, *F* value by permutation; SS, sum of squares; Unique perms, number of permutations run.

* Denotes a significant value of *p* ≤ 0.05.

** Denotes a significant value of *p* ≤ 0.01.

Further assessment with ANOSIM determined that at all taxonomic levels the communities in the control group were significantly different from those in the three chemical treatments (Figure 4, Table S9). Breaking down each chemical into their concentration treatments, all communities were significantly different from the controls except for M‐L and Ph‐H, but only the two Ph treatments were significantly different from each other (*p* = 0.018), as also shown in the PERMANOVA results (Figure 4, Table S7). Comparing the three chemical treatments to each other rather than the controls revealed two significantly different community pairs at phylum level with only one at family and at species level (Table S9). The communities in treatment Pe‐L were different to those in Ph‐L at phylum level (*p* = 0.048), with M‐H being different to Ph‐L at both phylum (*p* = 0.008) and family (*p* = 0.040) level, and Pe‐H being different to Ph‐H at family (*p* = 0.029) and species (*p* = 0.029) level (Table S9).

There was visible clustering of the control samples into two groups (Figure 4): replicates 1, 6, 8, and 9 (referred to as group 1) and replicates 2, 4, 5, 7, and 10 (referred to as group 2). Although the two clusters were relatively distinct from each other, their interactions with the spiked treatments as separate groups were almost identical to those as a single group. At phylum level, group 1 was significantly different from all spiked treatments and group 2 was significantly different from all spiked treatments except M‐L (near significant) (Table S10). At family level, group 1 was significantly different from all spiked treatments except M‐L (near significant) and Pe‐H (near significant), and group 2 was significantly different from all spiked treatments except M‐L. At species level, group 1 was significantly different from all spiked treatments except Pe‐H (near significant), and group 2 was significantly different from all spiked treatments except M‐L (Table S10).

### 3.5 Relationship Between Microbial Structure and Environmental Condition

**FIGURE 5:**
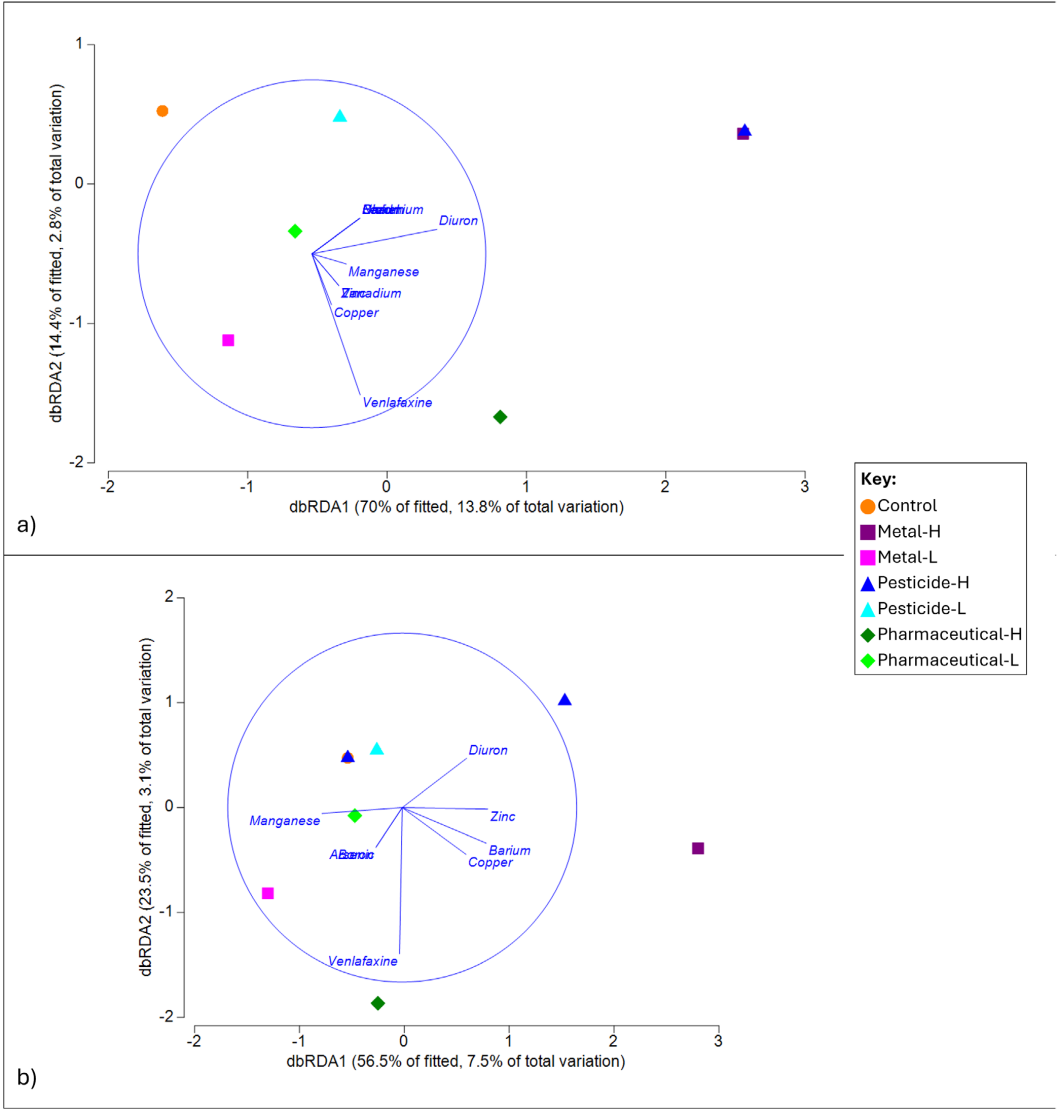
dbRDA plot of sediment (a) and water (b) chemical values and microbial communities for four treatments (control, metal, pesticide, pharmaceutical) and two concentrations (high, low). In Panel (a), M‐H and Pe‐H are plotted atop each other. In graph (b), C and Pe‐H are plotted atop each other.

The dbRDA ordination plots describe the relationship between chemical variables in the sediment and water and the variation in microbial communities at phylum level (Figure 5). Here, we define a positive correlation as a parallel relationship on one axis and a strong positive correlation as a parallel relationship on two axes. The first two dbRDA axes explain 16.6% of variation for the sediment (Figure 5a) and 10.6% of variation for the water (Figure 5b). In the sediment, 10 variables produced correlations with bacterial community structure (copper, lead, manganese, nickel, vanadium, zinc, barium, chromium, diuron, and venlafaxine), while there were only 8 in the water (copper, manganese, zinc, arsenic, barium, boron, diuron, and venlafaxine).

In the sediment, the communities in the M‐H samples were strongly positively correlated with lead, nickel, barium, and chromium, while the communities in the M‐L samples were strongly negatively correlated with lead, nickel, barium, and chromium (Figure 5a). The Pe‐H communities were strongly positively correlated with diuron, but the Pe‐L communities had no strong correlation. Similarly, the Ph‐H communities were strongly positively correlated with venlafaxine, while the Ph‐L communities had no strong correlation. The control communities were strongly negatively correlated with copper, manganese, vanadium, zinc, and venlafaxine (Figure 5a).

In the water, the M‐H communities were strongly positively correlated with copper, zinc, and barium, while the M‐L communities were strongly positively correlated with boron, arsenic, and manganese (Figure 5b). For the Pe‐H communities, only half of the replicates were strongly positively correlated with diuron, and the Pe‐L communities had no strong correlation. The Ph‐H and Ph‐L communities were all strongly positively correlated with venlafaxine. The control communities were strongly negatively correlated with copper, zinc, and barium (Figure 5b).

BEST analysis, which determines the best‐fit model for environmental values which best predict the distribution of the bacterial communities, of each treatment separately revealed that zinc and vanadium had the highest correlation value in sediment at 0.346, slightly above the rest of the metals, which sit equally at 0.343 (Table 2). In the water, barium and barium/copper combination produced the highest correlation at 0.343, slightly above copper (0.341) and the remainder of the metals (0.296). Diuron had a correlation value of 0.300 in sediment but only 0.012 in water, and venlafaxine had a correlation value of 0.161 in both sediment and water (Table 2).

**TABLE 2:** BEST analysis results for each treatment (metal, pesticide, pharmaceutical) for sediment and water.

No. Variables	Correlation	Variables
Sediment		
Metal		
1	0.346	Vanadium
1	0.346	Zinc
2	0.346	Vanadium, Zinc
3	0.343	Copper, Lead, Manganese
4	0.343	Copper, Lead, Manganese, Nickel
5	0.343	Copper, Lead, Manganese, Nickel, Vanadium
Pesticide		
1	0.300	Diuron
Pharmaceutical		
1	0.161	Venlafaxine
**Water**		
Metal		
1	0.341	Copper
1	0.343	Barium
2	0.343	Copper, Barium
3	0.269	Copper, Manganese, Zinc
4	0.269	Copper, Manganese, Zinc, Barium
5	0.269	Copper, Manganese, Zinc, Arsenic, Barium
Pesticide		
1	0.012	Diuron
Pharmaceutical		
1	0.161	Venlafaxine

*Note:* Metal results are displayed for the top five variables of a possible 15. Only one variable is available for pesticide and pharmaceutical results.

### 3.6 Taxonomic Variation Between Treatments

SIMPER analysis determined that at phylum level the control versus spiked (community) treatments had an average dissimilarity of ~7%, with the top five taxa which contributed most to this dissimilarity making up ~30% of the cumulative dissimilarity (Table S11). The same five phyla were present as the top five contributors across all pairwise comparisons: Bacteroidota, Cyanobacteria, Verrucomicrobiota, Actinobacteriota, and Proteobacteria. The spiked versus spiked treatments had an average dissimilarity of ~5% with the top five contributors making up ~25% of cumulative dissimilarity (Table S11).

At family level, the control versus spiked treatments had an average dissimilarity of ~14% with the top 10 contributors making up ~7% of cumulative dissimilarity (Table S12). Coleofasciculaceae, Nostocaceae, VadinHA17_877549 (a Bacteroidota family), Porticoccaceae, Prolixibacteraceae, JAAYJT01 (a Bacteroidota family), and UBA9973 (a Patescibacteria family) comprised the top seven contributors across all pairwise comparisons. The spiked versus spiked treatments had an average dissimilarity of ~12% with the top 10 contributors making up ~6% of cumulative dissimilarity. Nostocaceae, Coleofasciculaceae, Porticoccaceae, VadinHA17_877549, Bin106, and UBA9973 contributed to dissimilarity across all pairwise comparisons (Table S12).

At species level, control versus spiked treatments had an average dissimilarity of ~19% with the top 15 contributors making up ~4% of cumulative dissimilarity (Table S13). Only 11 of the 23 taxa that contributed to the control versus spiked dissimilarities could be identified to species. The spiked versus spiked treatments had an average dissimilarity of ~17% with the top 15 contributors making up ~4% of cumulative dissimilarity. Only eight of the 22 taxa which contributed to the spiked versus spiked dissimilarities could be identified to species (Table S13).

Overall, five phyla, 13 families, and three species were identified as potential bio‐indicators in this study. Of the phyla, Proteobacteria, Bacteroidota and Verrucomicrobiota responded negatively to contamination (reduction in relative abundance) while Cyanobacteria and Actinobacteriota responded positively to contamination (increase in relative abundance). The responses for all phyla were not significantly discernible between the three chemical classes (Table S11). Of the families, Prolixibacteraceae, Porticoccaceae, VadinHA17_877549 and UBA9973 responded negatively to contamination with no discernible responses between the chemicals. Lentimicrobiaceae, JAAYJT01 and UBA11358 responded negatively to contamination, more so for metals. Opitutaceae and UBA8199 responded negatively to contamination, more so for pharmaceuticals and pesticides, respectively. Nostocaceae responded negatively to contamination, for pesticide > pharmaceutical > metals. Coleofasciculaceae, Bin106 and Parachlamydiaceae responded positively to contamination. Coleofasciculaceae had no discernible responses between the chemicals, Bin106 responded more so for metals, and Parachlamydiaceae for pharmaceuticals and pesticides (Table S12). Of the species, *Nemorincola caseinilytica* and *Lentimicrobium saccharophilum* responded negatively to contamination and had no discernible responses between the chemicals. *Silvanigrella paludirubra* responded positively to contamination, more so for metals and pharmaceuticals (Table S13). The direction and specificity of responses for these taxa are summarised in Table 3.

**TABLE 3:** Summary of taxa showing pollutant treatment responses identified via SIMPER analysis with potential for use as pollutant indicators.

Taxa	Taxonomic rank	Response to contamination	Specific pollutant class?
Proteobacteria	Phylum	Negative*	Not discernible
Bacteroidota	Phylum	Negative*	Not discernible
Verrucomicrobiota	Phylum	Negative*	Not discernible
Cyanobacteria	Phylum	Positive*	Not discernible
Actinobacteriota	Phylum	Positive	Not discernible
Prolixibacteraceae	Family	Negative	Not discernible
Porticoccaceae	Family	Negative	Not discernible
VadinHA17_877549	Family	Negative	Not discernible
UBA9973	Family	Negative	Not discernible
Lentimicrobiaceae	Family	Negative	Metals
JAAYJT01	Family	Negative	Metals
UBA11358	Family	Negative	Metals
Opitutaceae	Family	Negative	Pharmaceutical
UBA8199	Family	Negative	Pesticide
Nostocaceae	Family	Negative	Pesticide>Pharmaceutical > Metals
Coleofasciculaceae	Family	Positive	Not discernible
Bin106	Family	Positive	Metals
Parachlamydiaceae	Family	Positive	Pesticide and Pharmaceutical
*Nemorincola caseinilytica*	Species	Negative	Not discernible
*Lentimicrobium saccharophilum*	Species	Negative	Not discernible
*Silvanigrella paludirubra*	Species	Positive	Metals and Pharmaceutical

* Indicates taxa which had responses that do not align with hypotheses/previous research.

### 3.7 Macroinvertebrate Analysis

Eighteen unique macroinvertebrate taxa (1825 individuals) were identified to the lowest possible taxonomic level (Table S14). Overall, abundances were too low, as well as variability between replicates being too high, to discern any statistically significant differences between treatments or to appropriately assess the variation of these macroinvertebrate taxa with a biotic index. Temperature and rainfall data for the experimental period are provided in Table S15.

## 4 Discussion

In this study, we investigated variation in wetland sediment bacterial community structure when challenged by three major contaminants. We sought to assess and compare the potential use of bacterial taxa (via eDNA analysis at three taxonomic levels) for application in microbial (bacterial)‐based endpoint‐analysis and bioindicator identification of pollutant impacts. We additionally compared this to a more established macroinvertebrate survey approach as a tool for exploring pollutant ecological impacts in wetland systems. It is important to understand how pollutants affect microbial communities given their key roles including carbon and nutrient cycling and pollutant bioremediation and, in turn, expand the set of techniques available to be used to provide comprehensive assessments of freshwater ecosystem health and condition (Pawlowski et al. 2018).

In these field microcosm experiments, five phyla were identified as contributing most to the variation in bacterial community structure between the control and spiked treatments, regardless of the chemical (Table 3). Proteobacteria are known to be widely tolerant of, and effective in, the removal of zinc (Chen et al. 2021) and copper (Yu et al. 2020); Bacteroidota have been associated with the removal of copper (Guo et al. 2021); Actinobacteriota have been found to be positively correlated with copper contamination (Lejon et al. 2008); and previous studies have shown Verrucomicrobiota to be tolerant of metals (Flynn et al. 2025; Zhu et al. 2023) and other urban pollutants (Flynn et al. 2025). Conversely, Cyanobacteria have widespread sensitivity to heavy metals (Flynn et al. 2025; Tchounwou et al. 2012) and pesticides (Flynn et al. 2025; Singh et al. 2018). Given the tolerances and sensitivities these phyla have shown (to metals in particular) in previous studies, it was expected that Cyanobacteria would be more abundant in the control samples while the spiked samples would have a higher abundance of Proteobacteria, Bacteroidota, Actinobacteriota, and Verrucomicrobiota. However, in our study only Actinobacteriota displayed the expected trend of having higher abundances in the pollutant‐treatment (spiked) samples, with the other four aforementioned phyla having relative abundances that were not expected from previous research.

We have identified 13 bacterial families (Bin106, Coleofasciculaceae, JAAYJT01, Lentimicrobiaceae, Nostocaceae, Opitutaceae, Parachlamydiaceae, Porticoccaceae, Prolixibacteraceae, UBA11358, UBA8199, UBA9973, and VadinHA17_877549) that exhibit potential to be used as bacterial bioindicators of aquatic contamination. Three families had a positive correlation to contamination (increase in relative abundance in the spiked treatments) while the other 10 had a negative relationship to contamination (decrease in relative abundance in the spiked treatments). Overall, five families were identified as indicating between a polluted versus nonpolluted environment, one family discriminated between metal versus non‐metal pollution, six families discriminated for a specific pollutant (four copper, one diuron, one venlafaxine), and one family was a potential indicator for all pollutants. The strength and direction for VadinHA17_877549 as a bioindicator in this study parallels those seen in previous studies from the same geographical area (Flynn et al., n.d.; Flynn et al. 2025). However, for Coleofasciculaceae the indication pattern is in contrast to what had been observed previously in the geographical area (Flynn et al., n.d.; Flynn et al. 2025), possibly due to increased overall productivity as a result of higher nutrient levels in the wetland (Bonilla et al. 2023; Feng et al. 2024). Of the eight families and one species identified as potential diagnostic bioindicators, eight taxa can be considered novel finds. JAAYJT01, UBA8199 and Bin106 currently have no published research looking into their function as bacterial bioindicators, outside of the current study. Opitutaceae has been found to be correlated with metal contamination (Fernández‐López et al. 2024), but the current study is the first to corelate it with pharmaceuticals. One study considered UBA11358 as a potential indicator to climate change related environmental variables (such as temperature, pH, and atmospheric pressure) and found no significant correlations (Penna et al. 2025), however, they did not assess its correlation to chemical contaminants as we have done. Likewise, Lentimicrobiaceae has been previously associated with physico‐chemical factors such as organic carbon content (Gao et al. 2023), and turbidity and nutrients (Li et al. 2024), but not chemical contaminants. There are only a few published studies which explore environmental occurrences of Parachlamydiaceae, with most research focusing on its potential parasitic nature within amoeba and humans (Corsaro and Venditti 2006). Similarly, *S. paludirubra* is a recently described species, one of only two in its family, with limited research available aside from its discovery and isolation from freshwater environments (Pitt et al. 2020). Nostocaceae has been associated with urbanisation (Fazlutdinova and Gaysina 2025), however, most studies consider only individual species. Given the limited publications available currently, further research into bio‐indicative ability for all the taxa described above is warranted.

Our results showed that the sediment‐associated diuron had a much greater correlation with bacterial structure than the water‐associated diuron, although both had less correlation when compared to the metals. A wide range of pesticides negatively affect soil and sedimentary bacterial communities, reducing diversity and activity (Jeyaseelan et al. 2024; Prado and Airoldi 2001). However, these effects were not always detectable through community‐level analysis (Widenfalk et al. 2008). Diuron, in particular, has been shown to cause a significant reduction in the rates of metabolism within soil bacterial communities (Prado and Airoldi 2001). The current study also showed that the correlation between venlafaxine and microbial community structure was the same for both the water and sediment samples. This venlafaxine correlation was greater than that of diuron in the water samples but lower than diuron in the sediment samples and lower than the correlations produced by the metals in both the sediment and water samples. Pharmaceuticals reduce activity (Menacherry et al. 2023) and alpha diversity in microbial communities (Shen et al. 2019). Venlafaxine, in particular, is known to resist natural degradation for a significant amount of time (Menacherry et al. 2023) but there is limited information describing its impact on sedimentary bacterial communities.

Despite copper being the only metal spiked into the metal treatment microcosms in this study, zinc, vanadium, barium, and copper had the greatest correlation to bacterial community structure. Given that copper binds strongly to sediments and particulate matter (ANZG 2018; Rader et al. 2019) it was not unexpected that other metals present in the wetland were released and became more bioavailable after the preferential binding of copper to the sediment (Flynn et al., n.d.; Wojtkowska 2013). At low concentrations zinc can be beneficial to the growth of bacteria (Molnar‐Nagy et al. 2022), but in high concentrations it can disrupt enzymatic activities and decrease microbial biomass (Molnar‐Nagy et al. 2022; Song et al. 2018). The influence of vanadium on sedimentary bacterial communities has received only limited attention, with the studies that do exist determining that it significantly shapes assemblage structure, generally increasing the proportion and diversity of metal tolerant taxa (Wang et al. 2022; Zhang et al. 2020). Copper is quite toxic to aquatic biota (ANZG 2018; Yang et al. 2018), with exposure causing reduced diversity (Lear et al. 2023; Mahamoud Ahmed et al. 2020), reduced productivity (Lear et al. 2023), and changes to functionality (Sutcliffe et al. 2019) in bacterial communities. Few studies have been published that assess the influence of barium on freshwater bacterial communities, but our previous study described a consistent high correlation of barium with bacterial assemblage structure with a probable negative correlation between barium concentration and bacterial diversity (Flynn et al. 2025), also seen in this study. The introduction of copper into the microcosm triggering a secondary release of other latent metals introduces additional complexity when determining what contaminants are having the greatest impact on microbial structures and assessing the overall impact of a copper pollution event. The confounding direct effects (copper toxicity) and indirect effects (zinc/barium/vanadium toxicity) seen in this study highlight a critical challenge faced in ecological assessments when attempting to predict or determine the impact a pollution event has had or will have on an aquatic ecosystem. Given the similar correlation factors seen in this study between copper, zinc, vanadium, and barium, it is likely that the observed microbial shifts are a result of multi‐metal interactions rather than copper alone.

Yachi and Loreau (1999) theorised that the more genetically diverse a community is the more stable it will be, as a result of the increased likelihood of taxa with resistance traits/genes being present in the community. This diversity can be a measure of an individual or combination of diversity indices including evenness (Wittebolle et al. 2009), richness (van Elsas et al. 2012) and biodiversity (Shade et al. 2012). The average biodiversity (as measured by Faith's PD) of the sediments used in this experiment from Glynns wetland were notably higher (around 6.5 times higher), than in other wetlands in the same geographical area which showed significant impacts of pollution on their microbial community structures (Flynn et al., n.d.; Flynn et al. 2025). Taxon evenness of Glynns wetland was also higher on average than for sediments in these previous studies (Flynn et al., n.d.; Flynn et al. 2025). The high diversity present in the wetland sediments in this study, as measured by biodiversity and evenness, compared to previous studies may account for the low variation of diversity seen between some of the samples, as well as the few taxa which did not display expected abundance trends in relation to pollutant inputs.

Comparing between the significant results returned at each taxonomic level, there were significant differences between the control and each chemical treatment at all taxonomic levels, although no clear trend was observed. The variations between concentration groups in each chemical treatment decreased with decreasing taxonomic level, but significant differences were still detectable down to species level. Correlations between bacterial structure and the environmental variables decreased slightly from phylum to species level. Given that no significant overall trends were seen with changing taxonomic level of analysis that were consistent across all treatments, and that at least one significant grouping was seen at all taxonomic levels, analyses of bacterial communities at either phylum, family, or species level will have value in assessing assemblage variation. When it comes to choosing a taxonomic level of analysis for ecosystem health assessments via community dynamics evaluations, all taxonomic levels should provide important insight into the responses of bacteria. Overall, phylum level analysis provided slightly more significant groupings and correlations in this study; however, if the aim of the analysis is to identify specific species and not community‐level variations, the ideal taxonomic level for assessment may be more impacted by the quality and taxon coverage of the taxonomic classifier used.

The capacity to identify individual species using a metabarcoding analysis relies on the ability of the classifier used to assign that taxonomy and which, in turn, relies on having a comprehensive reference database. The current version of the Greengenes2 classifier (v 2022.10), which was used in this study, loses significant resolution below the level of family. Ideally, when choosing taxa for a bioindicator index they should be at the lowest taxonomic level possible, taking into account the known tolerances of the taxa and the ability for users to identify taxa to that level. In theory, the lower the taxonomic level assessed, the less variation in tolerances will be present within the group (e.g., two taxa in the same family would be expected to have the same tolerance to a chemical, whereas two taxa that only share the same phylum could have some similarities but also some significant differences). Given the current limitations of microbial and bacterial classifiers in assigning taxonomy at lower taxonomic levels (a common constraint arising from the use of the 16S rRNA variable regions, affecting all classifiers including Greengenes (Bose and Moore 2023)), a bioindicator index at bacterial family level may be most useful, being the lowest possible taxonomic level at which identities can be confidently assigned for the majority of time.

In contrast to microbial community analysis, macroinvertebrate surveys are a wide‐spread and long‐established biomonitoring tool (Gieswein et al. 2019). However, the most commonly used and easily accessible macroinvertebrate indices in Australia are not suited to wetlands in southeast Australia and require a minimum number of total taxa and unique taxa to accurately provide an indication of environmental condition (Chessman 2001; Chessman 2003; Chessman et al. 2002; EPA Victoria 2010). Macroinvertebrate studies also generally rely on specialist expertise to accurately visually identify each individual, with only a few studies like Carew et al. (2018) using metabarcoding analysis for macroinvertebrate identification. In our study, the number and diversity of macroinvertebrates collected were not sufficient for use in an index, nor were any of the aforementioned indices appropriate for our study site or design. The lack of macroinvertebrate colonisation in the microcosms could be attributed to a larger than intended distance between the microcosms and water's edge, due to high temperatures and lower than expected rainfall over the duration of the study (BOM 2025). A benefit of eDNA analysis over macroinvertebrate surveys is the elimination of the minimum number of taxa requirement barrier; in contrast, eDNA analysis of sediments will yield very high numbers of sequence reads (Ellegaard et al. 2020) with the benefit also of removing the need for specialist visual expertise for identification, as taxonomy is assigned with a classifier.

While our study highlights strong acute microbial structural responses to contamination, it is important to note that the temporal limitations that arise from a microcosm study are somewhat opposed to the long‐term stability which is generally required in ecological monitoring assessments. Although the novel bioindicators highlighted in our study showed good potential to indicate a more recent contamination event, more research is required to determine if these responses will remain detectable months after exposure or if the communities will recover to their baseline state. Additionally, integrating functional genomic analyses (such as metagenomics or metabolomics) into a structural assessment could further support the applicability of potential bioindicators by identifying the genes responsible for tolerance along with the taxa in which they reside, as well as assessing the functional consequences that result from a shift in community structure which in turn will aid in understanding and assessing the overall health and performance of a wetland. Whilst this study shows promising results of potential microbial bioindicators, we feel that further validation experiments focusing on responses from combined stressor effects and efforts to benchmark baseline microbial fluctuations for different sediment types and environments are the appropriate next steps to work towards integrating microbial eDNA analyses into existing regulatory frameworks alongside traditional biological indices.

## 5 Conclusion

In this study, we described the impact of three major contaminants on wetland ecosystems by assessing their effects on sediment microbial communities, comparing the responses between the contaminants, between taxonomic levels and also to their macroinvertebrate community. Our analysis showed that five phyla (Proteobacteria, Bacteroidota, Actinobacteriota, Verrucomicrobiota, Cyanobacteria) were responsible for the majority of variation between the control and spiked treatments, regardless of which chemical was present, and we identified 13 bacterial families which have the potential to be used diagnostically to differentiate between different classes of pollutant, eight of which are novel bioindicators. We found that diuron and venlafaxine had similar effects on bacterial structures, which were less than that of metals, where we saw that zinc, vanadium, and barium had high correlations to bacterial structure as well as the spiked copper. We suggest that bacterial indices should be created at the lowest taxonomic level at which a classifier can confidently assign taxonomy most of the time and that the taxonomic level of analysis in eDNA studies should be determined by the aim of the study and the level of reliable resolution of the taxonomic classifier being used. We also suggest that microbial eDNA analysis should be included in the suite of biomonitoring tools for wetland health assessments, alongside macroinvertebrate assessments, as it produces a larger data set overall, eliminates the need for expertise when assigning taxonomy, and is suitable for environments where macroinvertebrate indices do not otherwise exist. This study demonstrated the impacts of chemical contamination on freshwater wetlands by characterising the responses of sedimentary microbial communities to these chemicals.

## Author Contributions

**A. S. Flynn:** conceptualization, investigation, writing – review and editing, writing – original draft, methodology, formal analysis. **A. M. Osborn:** conceptualization, methodology, writing – review and editing. **V. Pettigrove:** conceptualization, writing – review and editing, methodology. **J. Shimeta:** conceptualization, methodology, writing – review and editing. **S. M. Long:** conceptualization, writing – review and editing, methodology.

## Funding

This work was supported by Melbourne Water. Department of Education, Australian Government.

## Conflicts of Interest

The authors declare no conflicts of interest.

## Supporting information


**Data S1:** Supporting Information.
**Table S1:** Glynn's wetland preliminary sediment chemistry results. ANZG (2018) sediment default guideline values (DGV) and high guideline values (HGV) are included where available. All measured pesticides were below limit of detection. TPH = Total Petroleum Hydrocarbons.
**Table S2:** Randomly assigned microcosm number for each sample replicate. C = control, M = metal treatment, Ph = pharmaceutical treatment, Pe = pesticide treatment. L = low dose, H = high dose.
**Table S3:** Details of spiking solution stock concentrations and spiking volumes. C = control, M = metal treatment, Ph = pharmaceutical treatment, Pe = pesticide treatment. L = low dose, H = high dose.
**Table S4:** Freshwater 95% and 80% trigger value (TV) for copper (ANZG 2018) and diuron (King et al. 2017). Sediment default guideline value (DGV) and high guideline value (HGV) for copper (ANZG 2018). No sediment guideline data is available for diuron.
**Table S5a:** Metal concentrations for mercury (Hg), chromium (Cr), cobalt (Co), lead (Pb), manganese (Mn), arsenic (As), nickel (Ni), selenium (Se), beryllium (Be), vanadium (V), zinc (Zn), barium (Ba), boron (B), and cadmium (Cd) measured in water samples, including limits of detection (LoD) and ANZG (2018) guideline values, where available.
**Table S5b:** Metal concentrations for mercury (Hg), chromium (Cr), cobalt (Co), lead (Pb), manganese (Mg), arsenic (As), nickel (Ni), selenium (Se), beryllium (Be), vanadium (V), zinc (Zn), barium (Ba), boron (B), and cadmium (Cd) measured in sediment samples, including limits of detection (LoD) and ANZG (2018) guideline values, where available.
**Table S6:** Alpha diversity metrics (evenness, Shannon diversity, Chao1, Faith's PD) ANOVAs for each treatment (metal, pesticide, pharmaceutical). * denotes a significant value of *p* ≤ 0.05.
**Table S7a:** Pairwise treatment group PERMANOVA results for phylum, family, and species. * denotes a significant value of *p* ≤ 0.05 and ** denotes a significant value of *p* ≤ 0.01. Perm = permutation.
**Table S7b:** Pairwise concentration group PERMANOVA results for phylum, family, and species. * denotes a significant value of *p* ≤ 0.05. Perm = permutation.
**Table S8a:** Pairwise treatment group PERMANOVA results for ASV data (global t = 1.8645, *p* = 0.032, 999 permutations). * denotes a significant value of *p* ≤ 0.05. Perm = permutation.
**Table S8b:** Pairwise treatment group PERMANOVA results for ASV data (global t = 1.4894, *p* = 0.042, 999 permutations). * denotes a significant value of p ≤ 0.05. Perm = permutation.
**Table S9:** Pairwise ANOSIM results comparing (a) treatment groups and (b) treatment‐concentration groups at phylum, family, and species level. For table (a) * denotes a significant value of *p* ≤ 0.05 and ** denotes a significant value of *p* ≤ 0.01. For table (b) only significant results of p ≤ 0.05 are shown. M = metal treatment, Ph = pharmaceutical treatment, Pe = pesticide treatment. L = low dose, H = high dose.
**Table S10:** Separate control group versus treatment‐concentration group pairwise ANOSIM results. * denotes a significant value of *p* ≤ 0.05. C1 = control group 1, C2 = control group 2. L = low dose, H = high dose.
**Table S11:** SIMPER analysis results comparing phyla between treatments. Average abundance (Av. Abun), contribution (Cont. %) and cumulative contribution (Cum. %) are shown for the top five contributing taxa.
**Table S12:** SIMPER analysis results comparing families between treatments. Average abundance (Av. Abun), contribution (Cont. %) and cumulative contribution (Cum. %) are shown for the top 10 contributing taxa. Taxa that could not be classified down to family are written as “Phylum; lowest possible classification; f__”. C = control, M = metal treatment, Ph = pharmaceutical treatment, Pe = pesticide treatment.
**Table S13:** SIMPER analysis results comparing species between treatments. Average abundance (Av. Abun), contribution (Cont. %) and cumulative contribution (Cum. %) are shown for the top 15 contributing taxa. Taxa that could not be classified down to species are written as “Phylum; lowest possible classification; s__”. C = control, M = metal treatment, Ph = pharmaceutical treatment, Pe = pesticide treatment.
**Table S14:** Macroinvertebrate abundance table. C = control, M = metal treatment, Ph = pharmaceutical treatment, Pe = pesticide treatment. L = low dose, H = high dose.
**Table S15:** Daily temperatures and rainfall over the duration of microcosm deployment, and averages for those months (BOM 2025). OCT = October, NOV = November.
**Table S16:** Water quality measurement results from weeks 1 (deployment), 2, and 3 of the 4 week experiment. No data was obtained at week 4 (conclusion) due to malfunctions with the field equipment. C = control, M = metal treatment, Ph = pharmaceutical treatment, Pe = pesticide treatment. L = low dose, H = high dose.

## Data Availability

The sequence data have been submitted to the Sequence Read Archive (SRA) databases under accession number PRJNA1297592.
