# Supplementary material for: Aquatic Bacterial Community Responses to Aquatic Contaminants Revealed by 16S rRNA Metabarcoding in Field‐Based Microcosms
Source: Environ Microbiol Rep. 2026 Mar 27;18(2):e70328. doi: 10.1111/1758-2229.70328 (PMC13140830; doi:10.1111/1758-2229.70328)
Supplement: Supplementary file 1 — Data S1: Supporting Information. Table S1: Glynn's wetland preliminary sediment chemistry results. ANZG (2018) sediment default guideline values (DGV) and high guideline values (HGV) are included where available. All measured pesticides were below limit of detection. TPH = Total Petroleum Hydrocarbons. Table S2: Randomly assigned microcosm number for each sample replicate. C = control, M = metal treatment, Ph = pharmaceutical treatment, Pe = pesticide treatment. L = low dose, H = high dose. Table S3: Details of spiking solution stock concentrations and spiking volumes. C = control, M = metal treatment, Ph = pharmaceutical treatment, Pe = pesticide treatment. L = low dose, H = high dose. Table S4: Freshwater 95% and 80% trigger value (TV) for copper (ANZG 2018) and diuron (King et al. 2017). Sediment default guideline value (DGV) and high guideline value (HGV) for copper (ANZG 2018). No sediment guideline data is available for diuron. Table S5a: Metal concentrations for mercury (Hg), chromium (Cr), cobalt (Co), lead (Pb), manganese (Mn), arsenic (As), nickel (Ni), selenium (Se), beryllium (Be), vanadium (V), zinc (Zn), barium (Ba), boron (B), and cadmium (Cd) measured in water samples, including limits of detection (LoD) and ANZG (2018) guideline values, where available. Table S5b: Metal concentrations for mercury (Hg), chromium (Cr), cobalt (Co), lead (Pb), manganese (Mg), arsenic (As), nickel (Ni), selenium (Se), beryllium (Be), vanadium (V), zinc (Zn), barium (Ba), boron (B), and cadmium (Cd) measured in sediment samples, including limits of detection (LoD) and ANZG (2018) guideline values, where available. Table S6: Alpha diversity metrics (evenness, Shannon diversity, Chao1, Faith's PD) ANOVAs for each treatment (metal, pesticide, pharmaceutical). * denotes a significant value of p ≤ 0.05. Table S7a: Pairwise treatment group PERMANOVA results for phylum, family, and species. * denotes a significant value of p ≤ 0.05 and ** denotes a significant value of p ≤ 0.01 [file EMI4-18-e70328-s001.docx]

**Freshwater bacterial community responses to aquatic contaminants revealed by 16S rRNA metabarcoding in field-based microcosms**

**Flynn, A.S., Osborn, A.M., Pettigrove, V., Shimeta, J. and Long, S.M.**

Method S1: Pharmaceutical and Personal care Products (PPCPs) testing in water – Venlafaxine in sediment and water) by National Measurement Institute (NMI) (Port Melbourne, Australia).

Sample Preparation and Extraction

For sample preparation, 30 mL of Milli-Q water was dispensed into a 50 mL plastic centrifuge tube for use as blanks and spikes, while 30 mL of pre-filtered sample was added to a separate 50 mL tube. Spiking was conducted at three concentration levels (1,5 and 20ug/L). All tubes were vortexed and shaken vigorously on a horizontal shaker for 5 minutes to ensure thorough mixing. Prepare blank cartridges by conditioning them on a vacuum manifold, first with 8 mL of methanol, then with 8 mL of Milli-Q water, using gravity only. Load the blank, spiked, and sample extracts onto the cartridges under low vacuum, keeping the flow rate at 1–2 drops per second. After loading, dry the cartridges and elute each one with two portions of 2.5 mL methanol, followed by 2.5 mL Milli-Q water. Dry the cartridges again under high vacuum. Then re-elute with 2.5 mL methanol and 1.5 mL Milli-Q water. Finish by drying all cartridges under high vacuum for 30 seconds. Reduce the final volume to 4 mL using a constant stream of nitrogen at 45 °C on the reactivetherm. Once dry, add 1 mL of acetonitrile and top up to 5 mL with Milli-Q water. Mix the extract by vortexing and filter it through a 0.45 μm disc filter into a 4 mL amber vial. For LC-MS/MS analysis, add 5 μL of mixed internal standard to 495 μL of sample in Kimble glass tubes. Vortex to mix, then transfer the final extracts into amber LC-MS/MS vials for analysis.

Instrumentation and Software and Quality control

A Waters TQS Tandem Quadrupole Detector Liquid Chromatograph-Mass Spectrometer (LC-MS/MS) and an ACQUITY UPLC BEH C18 100 × 2.1 mm column were used for detection and quantify concentrations of each Pharmaceutical and Personal care Products (PPCPs). For each analytical batch, a matrix blank, solvent blank, seven points (0.0ug/L – 200 ug/L) matrix matched calibration and 3 spikes levels (1,2, and 20 ug/L) were performed to ensure all the required QA and QC are met for the reportable results. Duplicate results were performed for every 10th samples. Recovery rates for each Pharmaceutical and Personal care Products (PPCPs), were calculated using samples matrix spiked with analytical standards. The limits of detection (LOD) were generally half of Level of Reporting (LOR) ie.0.5 ug/L for most of compounds. Values below the LOR and above the LOD are reported as TRACE detections indicating presence but at low concentrations.

The extract components were separated on a Acquity UPLC BEH C18 column (2.1 mm × 100 mm; 1.7 μm) maintained at 40 °C with a Vanguard. Using a gradient of 0.1% formic Acid in Water (Solvent A) and 0.1% formic Acid in Acetonitrile (Solvent B). The sample components were loaded on to the column (0.2mL/min) with a starting gradient of 95 % solvent A maintained for 2 min, followed by 85 % solvent A from2 to 4min, then 30 % solvent A from 4 to 6 min, followed by 5 % solvent A from 6.5 min to 7.0 min, and ﬁnally increase to 95 % solvent A until 10 min. All target compounds were eluted from 2.1 min to 7.6 min and the total runtime was 10 min. The auto sampler was operated at 10 °C ± 5°C. Data acquisition and processing were conducted using MassLynx 4.2 and Target Lynx XS software (Waters Corporation).

(Note: Venlafaxine [elution @ 6.56](mailto:elution@6.56) min)

Table S1. Glynn's wetland preliminary sediment chemistry results. ANZG (2018) sediment default guideline values (DGV) and high guideline values (HGV) are included where available. All measured pesticides were below limit of detection. TPH = Total Petroleum Hydrocarbons.

|  |  | **Metals (mg/kg)** | | | | | | | | | | | | | | | **TPH (mg/kg)** | **Total Organic Carbon (%)** |
| --- | --- | --- | --- | --- | --- | --- | --- | --- | --- | --- | --- | --- | --- | --- | --- | --- | --- | --- |
| **Site** | **Date** | **Arsenic** | **Barium** | **Beryllium** | **Boron** | **Cadmium** | **Chromium (III+VI)** | **Cobalt** | **Copper** | **Lead** | **Manganese** | **Mercury** | **Nickel** | **Selenium** | **Vanadium** | **Zinc** | **C10-C36 Fraction (Sum)** | **Total Organic Carbon** |
| **Glynns** | **26/08/24** | <5 | 150 | <1 | <50 | <1 | 24 | 5 | 10 | 14 | 102 | <0.1 | 13 | <5 | 25 | 50 | 620 | 3.06 |
| ANZG (2018) DGV | | 20 | - | - | - | 1.5 | 80 | - | 65 | 50 | - | 0.15 | 21 | - | - | 200 | - | - |
| ANZG (2018) HGV | | 70 | - | - | - | 10 | 370 | - | 270 | 220 | - | 1 | 52 | - | - | 410 | - | - |

Table S2. Randomly assigned microcosm number for each sample replicate. C = control, M = metal treatment, Ph = pharmaceutical treatment, Pe = pesticide treatment. L = low dose, H = high dose.

| **Sample (treatment-dose-replicate)** | **Microcosm number** |
| --- | --- |
| M-L-3 | 1 |
| Ph-L-2 | 2 |
| M-H-1 | 3 |
| Ph-L-4 | 4 |
| Pe-L-3 | 5 |
| M-H-2 | 6 |
| C-7 | 7 |
| C-1 | 8 |
| C-4 | 9 |
| C-10 | 10 |
| Pe-L-4 | 11 |
| Ph-L-5 | 12 |
| Pe-H-5 | 13 |
| C-14 | 14 |
| C-5 | 15 |
| Pe-H-3 | 16 |
| C-12 | 17 |
| M-L-2 | 18 |
| Ph-H-2 | 19 |
| C-15 | 20 |
| Ph-H-3 | 21 |
| M-H-3 | 22 |
| C-3 | 23 |
| M-L-4 | 24 |
| Ph-L-3 | 25 |
| Pe-H-2 | 26 |
| M-H-5 | 27 |
| Pe-L-2 | 28 |
| Pe-L-5 | 29 |
| M-L-5 | 30 |
| C-11 | 31 |
| Pe-H-1 | 32 |
| C-9 | 33 |
| Pe-H-4 | 34 |
| Ph-H-1 | 35 |
| Ph-H-4 | 36 |
| M-L-1 | 37 |
| Ph-L-1 | 38 |
| M-H-4 | 39 |
| Pe-L-1 | 40 |
| C-6 | 41 |
| C-13 | 42 |
| C-8 | 43 |
| Ph-H-5 | 44 |
| C-2 | 45 |

Table S3. Details of spiking solution stock concentrations and spiking volumes. C = control, M = metal treatment, Ph = pharmaceutical treatment, Pe = pesticide treatment. L = low dose, H = high dose.

| **Treatment** | **Spiking solution** | **Concentration (mg/L)** | **Volume added (mL)** | **Final concentration (µg/L)** |
| --- | --- | --- | --- | --- |
| C | Site water | - | 10 | - |
| M-L | CuSO_4_ | 10 copper | 42 | 28 |
| M-H |  |  | 75 | 50 |
| Pe-L | C_9_H_10_Cl_2_N_2_O | 10 diuron | 7.5 | 5 |
| Pe-H |  |  | 27 | 18 |
| Ph-L | C_17_H_27_NO_2_· HCl | 10 venlafaxine | 28.5 | 19 |
| Ph-H |  |  | 90 | 60 |

Table S4. Freshwater 95% and 80% trigger value (TV) for copper (ANZG 2018) and diuron (King et al. 2017). Sediment default guideline value (DGV) and high guideline value (HGV) for copper (ANZG 2018). No sediment guideline data is available for diuron.

| **Freshwater (µg/L)** | | |
| --- | --- | --- |
|  | **95% TV** | **80% TV** |
| Copper | 1.4 | 2.5 |
| Diuron | 0.23 | 0.9 |
| **Sediment (mg/kg)** | | |
|  | **DGV** | **HGV** |
| Copper | 65 | 270 |
| Diuron | - | - |

Table S5a. Metal concentrations for mercury (Hg), chromium (Cr), cobalt (Co), lead (Pb), manganese (Mn), arsenic (As), nickel (Ni), selenium (Se), beryllium (Be), vanadium (V), zinc (Zn), barium (Ba), boron (B), and cadmium (Cd) measured in water samples, including limits of detection (LoD) and ANZG (2018) guideline values, where available.

|  | **Hg** | **Cr** | **Co** | **Pb** | **Mn** | **As** | **Ni** |
| --- | --- | --- | --- | --- | --- | --- | --- |
|  | µg/L | | | | | | |
| **M-H** | <LoD | 1 | <LoD | <LoD | 3 | <LoD | <LoD |
| **M-L** | <LoD | 1 | <LoD | <LoD | 5 | 1 | <LoD |
| **C** | <LoD | 1 | <LoD | <LoD | 4 | <LoD | <LoD |
| **LoD** | 0.1 | 1 | 1 | 1 | 1 | 1 | 1 |
| **95% TV** | - | 1 | - | 3.4 | - | 13 | 11 |
| **80% TV** | - | 40 | - | 9.4 | - | 140 | 17 |
|  | **Se** | **Be** | **V** | **Zn** | **Ba** | **B** | **Cd** |
|  | µg/L | | | | | | |
| **M-H** | <LoD | <LoD | <LoD | 18 | 14 | <LoD | <LoD |
| **M-L** | <LoD | <LoD | <LoD | 6 | 11 | 50 | <LoD |
| **C** | <LoD | <LoD | <LoD | 11 | 10 | <LoD | <LoD |
| **LoD** | 10 | 1 | 10 | 5 | 1 | 5 | 0.1 |
| **95% TV** | - | - | - | 8 | - | - | 0.2 |
| **80% TV** | - | - | - | 31 | - | - | 0.8 |

Table S5b. Metal concentrations for mercury (Hg), chromium (Cr), cobalt (Co), lead (Pb), manganese (Mg), arsenic (As), nickel (Ni), selenium (Se), beryllium (Be), vanadium (V), zinc (Zn), barium (Ba), boron (B), and cadmium (Cd) measured in sediment samples, including limits of detection (LoD) and ANZG (2018) guideline values, where available.

|  | **Hg** | **Cr** | **Co** | **Pb** | **Mg** | **As** | **Ni** |
| --- | --- | --- | --- | --- | --- | --- | --- |
|  | mg/kg | | | | | | |
| **M-H** | 0 | 26 | 6 | 16 | 117 | 0 | 14 |
| **M-L** | 0 | 25 | 6 | 15 | 113 | 0 | 13 |
| **C** | 0 | 25 | 6 | 15 | 111 | 0 | 13 |
| **LoD** | 0.1 | 2 | 2 | 5 | 5 | 5 | 2 |
| **DGV** | 0.15 | 80 | - | 50 | - | 20 | 21 |
| **HGV** | 1 | 370 | - | 220 | - | 70 | 52 |
|  | **Se** | **Be** | **V** | **Zn** | **Ba** | **B** | **Cd** |
|  | mg/kg | | | | | | |
| **M-H** | 0 | 0 | 27 | 53 | 160 | 0 | 0 |
| **M-L** | 0 | 0 | 26 | 52 | 150 | 0 | 0 |
| **C** | 0 | 0 | 25 | 51 | 150 | 0 | 0 |
| **LoD** | 5 | 1 | 1 | 5 | 1 | 50 | 1 |
| **DGV** | - | - | - | 200 | - | - | 1.5 |
| **HGV** | - | - | - | 410 | - | - | 10 |

Table S6. Alpha diversity metrics (evenness, Shannon diversity, Chao1, Faith’s PD) ANOVAs for each treatment (metal, pesticide, pharmaceutical). * denotes a significant value of p ≤ 0.05.

| **Evenness** | | | | | |
| --- | --- | --- | --- | --- | --- |
| **Metal** | | | | | |
| Source | DF | Adj SS | Adj MS | F-Value | P-Value |
| Treatment | 2 | 0.000125 | 0.000063 | 0.85 | 0.444 |
| Error | 16 | 0.001173 | 0.000073 |  |  |
| Total | 18 | 0.001298 |  |  |  |
| **Pesticide** | | | | | |
| Source | DF | Adj SS | Adj MS | F-Value | P-Value |
| Treatment | 2 | 0.000696 | 0.000348 | 2.07 | 0.157 |
| Error | 17 | 0.002859 | 0.000168 |  |  |
| Total | 19 | 0.003554 |  |  |  |
| **Pharmaceutical** | | | | | |
| Source | DF | Adj SS | Adj MS | F-Value | P-Value |
| Treatment | 2 | 0.00006 | 0.00003 | 0.36 | 0.701 |
| Error | 16 | 0.001311 | 0.000082 |  |  |
| Total | 18 | 0.001371 |  |  |  |
| **Shannon** | | | | | |
| **Metal** | | | | | |
| Source | DF | Adj SS | Adj MS | F-Value | P-Value |
| Treatment | 2 | 0.0211 | 0.01055 | 0.86 | 0.44 |
| Error | 16 | 0.19535 | 0.01221 |  |  |
| Total | 18 | 0.21645 |  |  |  |
| **Pesticide** | | | | | |
| Source | DF | Adj SS | Adj MS | F-Value | P-Value |
| Treatment | 2 | 0.07295 | 0.03648 | 1.7 | 0.212 |
| Error | 17 | 0.36409 | 0.02142 |  |  |
| Total | 19 | 0.43704 |  |  |  |
| **Pharmaceutical** | | | | | |
| Source | DF | Adj SS | Adj MS | F-Value | P-Value |
| Treatment | 2 | 0.006242 | 0.003121 | 0.25 | 0.782 |
| Error | 16 | 0.199804 | 0.012488 |  |  |
| Total | 18 | 0.206046 |  |  |  |
| **Biodiversity (Faith’s PD)** | | | | | |
| **Metal** | | | | | |
| Source | DF | Adj SS | Adj MS | F-Value | P-Value |
| Treatment | 2 | 4.74 | 2.37 | 0.27 | 0.768 |
| Error | 16 | 141.343 | 8.834 |  |  |
| Total | 18 | 146.083 |  |  |  |
| **Pesticide** | | | | | |
| Source | DF | Adj SS | Adj MS | F-Value | P-Value |
| Treatment | 2 | 2.711 | 1.355 | 0.18 | 0.833 |
| Error | 17 | 125.078 | 7.358 |  |  |
| Total | 19 | 127.789 |  |  |  |
| **Pharmaceutical** | | | | | |
| Source | DF | Adj SS | Adj MS | F-Value | P-Value |
| Treatment | 2 | 1.338 | 0.6691 | 0.07 | 0.929 |
| Error | 16 | 145.695 | 9.1059 |  |  |
| Total | 18 | 147.033 |  |  |  |

Table S7a. Pairwise treatment group PERMANOVA results for phylum, family, and species. * denotes a significant value of p ≤ 0.05 and ** denotes a significant value of p ≤ 0.01. Perm = permutation.

| **Phylum** | | | |
| --- | --- | --- | --- |
| Groups | t | P (perm) | Unique perms |
| Control, Metal | 1.7748 | 0.026* | 998 |
| Control, Pesticide | 2.2404 | 0.001** | 999 |
| Control, Pharmaceutical | 2.0405 | 0.005** | 999 |
| Metal, Pesticide | 0.83001 | 0.665 | 999 |
| Metal, Pharmaceutical | 0.77954 | 0.76 | 999 |
| Pesticide, Pharmaceutical | 0.81764 | 0.686 | 998 |
| **Family** | | | |
| Groups | t | P (perm) | Unique perms |
| Control, Metal | 1.3574 | 0.053 | 996 |
| Control, Pesticide | 1.7403 | 0.002** | 997 |
| Control, Pharmaceutical | 1.5558 | 0.005** | 995 |
| Metal, Pesticide | 1.0454 | 0.263 | 997 |
| Metal, Pharmaceutical | 0.93983 | 0.6 | 999 |
| Pesticide, Pharmaceutical | 0.95019 | 0.57 | 997 |
| **Species** | | | |
| Groups | t | P (perm) | Unique perms |
| Control, Metal | 1.282 | 0.055 | 997 |
| Control, Pesticide | 1.6091 | 0.002** | 999 |
| Control, Pharmaceutical | 1.4237 | 0.006** | 996 |
| Metal, Pesticide | 1.1003 | 0.153 | 997 |
| Metal, Pharmaceutical | 0.97072 | 0.533 | 999 |
| Pesticide, Pharmaceutical | 0.98033 | 0.527 | 998 |

Table S7b. Pairwise concentration group PERMANOVA results for phylum, family, and species. * denotes a significant value of p ≤ 0.05. Perm = permutation.

| **Phylum** | | | |
| --- | --- | --- | --- |
| Groups | t | P (perm) | Unique perms |
| High, Low (within “Metal”) | 1.3971 | 0.14 | 35 |
| High, Low (within “Pesticide”) | 1.6306 | 0.058 | 126 |
| High, Low (within “Pharmaceutical”) | 1.9876 | 0.023* | 56 |
| **Family** | | | |
| Groups | t | P (perm) | Unique perms |
| High, Low (within “Metal”) | 1.1725 | 0.141 | 35 |
| High, Low (within “Pesticide”) | 1.2076 | 0.132 | 126 |
| High, Low (within “Pharmaceutical”) | 1.3674 | 0.019* | 56 |
| **Species** | | | |
| Groups | t | P (perm) | Unique perms |
| High, Low (within “Metal”) | 1.1293 | 0.161 | 35 |
| High, Low (within “Pesticide”) | 1.1887 | 0.086 | 126 |
| High, Low (within “Pharmaceutical”) | 1.2614 | 0.021* | 56 |

Table S8a. Pairwise treatment group PERMANOVA results for ASV data (global t = 1.8645, p = 0.032, 999 permutations). * denotes a significant value of p ≤ 0.05. Perm = permutation.

| Groups | t | P (perm) | Unique perms |
| --- | --- | --- | --- |
| Control, Metal | 1.7967 | 0.109 | 999 |
| Control, Pesticide | 3.4674 | 0.014* | 999 |
| Control, Pharmaceutical | 2.9932 | 0.020* | 999 |
| Metal, Pesticide | 0.9971 | 0.381 | 999 |
| Metal, Pharmaceutical | 0.6268 | 0.727 | 999 |
| Pesticide, Pharmaceutical | 0.6281 | 0.737 | 999 |

Table S8b. Pairwise treatment group PERMANOVA results for ASV data (global t = 1.4894, p = 0.042, 999 permutations). * denotes a significant value of p ≤ 0.05. Perm = permutation.

| Groups | t | P (perm) | Unique perms |
| --- | --- | --- | --- |
| High, Low (within “Metal”) | 0.9764 | 0.436 | 999 |
| High, Low (within “Pesticide”) | 1.3590 | 0.240 | 999 |
| High, Low (within “Pharmaceutical”) | 1.4859 | 0.147 | 999 |

Table S9. Pairwise ANOSIM results comparing (a) treatment groups and (b) treatment-concentration groups at phylum, family, and species level. For table (a) * denotes a significant value of p ≤ 0.05 and ** denotes a significant value of p ≤ 0.01. For table (b) only significant results of p ≤ 0.05 are shown. M = metal treatment, Ph = pharmaceutical treatment, Pe = pesticide treatment. L = low dose, H = high dose.

| \| **(a)** \|  \|  \|  \| \| --- \| --- \| --- \| --- \| \| **Phylum** \|  \|  \|  \| \| Group 1 \| Group 2 \| R statistic \| p-value \| \| Control \| Pe \| 0.416 \| 0.002** \| \| Control \| Ph \| 0.433 \| 0.003** \| \| Control \| M \| 0.307 \| 0.013* \| \| M \| Ph \| 0.006 \| 0.404 \| \| M \| Pe \| -0.077 \| 0.802 \| \| Pe \| Ph \| -0.181 \| 1 \| \| **Family** \|  \|  \|  \| \| Group 1 \| Group 2 \| R statistic \| p-value \| \| Control \| Pe \| 0.453 \| 0.001** \| \| Control \| Ph \| 0.425 \| 0.002** \| \| Control \| M \| 0.238 \| 0.018* \| \| M \| Ph \| 0.064 \| 0.202 \| \| M \| Pe \| 0.044 \| 0.292 \| \| Pe \| Ph \| -0.107 \| 0.963 \| \| **Species** \|  \|  \|  \| \| Group 1 \| Group 2 \| R statistic \| p-value \| \| Control \| Pe \| 0.393 \| 0.001** \| \| Control \| Ph \| 0.466 \| 0.001** \| \| Control \| M \| 0.215 \| 0.047* \| \| M \| Ph \| 0.133 \| 0.104 \| \| M \| Pe \| 0.011 \| 0.378 \| \| Pe \| Ph \| -0.066 \| 0.802 \| | \| **(b)** \|  \|  \|  \| \| --- \| --- \| --- \| --- \| \| **Phylum** \|  \|  \|  \| \| Group 1 \| Group 2 \| R statistic \| p-value \| \| Control \| Ph-L \| 0.51 \| 0.006 \| \| Control \| M-H \| 0.489 \| 0.006 \| \| M-H \| Ph-L \| 0.475 \| 0.008 \| \| Control \| Pe-H \| 0.462 \| 0.008 \| \| Ph-H \| Ph-L \| 0.877 \| 0.018 \| \| Control \| Pe-L \| 0.35 \| 0.023 \| \| Pe-L \| Ph-L \| 0.272 \| 0.048 \| \| **Family** \|  \|  \|  \| \| Group 1 \| Group 2 \| R statistic \| p-value \| \| Control \| Ph-L \| 0.424 \| 0.003 \| \| Control \| Pe-H \| 0.377 \| 0.013 \| \| Ph-H, \| Ph-L \| 0.641 \| 0.018 \| \| Control \| M-H \| 0.393 \| 0.018 \| \| Control \| Pe-L \| 0.326 \| 0.023 \| \| Pe-H \| Ph-H \| 0.537 \| 0.029 \| \| M-H \| Ph-L \| 0.394 \| 0.040 \| \| **Species** \|  \|  \|  \| \| Group 1 \| Group 2 \| R statistic \| p-value \| \| Control \| Ph-L \| 0.382 \| 0.002 \| \| Control \| Pe-L \| 0.358 \| 0.010 \| \| Control \| Pe-H \| 0.352 \| 0.017 \| \| Ph-H \| Ph-L \| 0.621 \| 0.018 \| \| Control \| M-H \| 0.368 \| 0.020 \| \| Pe-H \| Ph-H \| 0.611 \| 0.029 \| |
| --- | --- | --- | --- | --- | --- | --- | --- | --- | --- | --- | --- | --- | --- | --- | --- | --- | --- | --- | --- | --- | --- | --- | --- | --- | --- | --- | --- | --- | --- | --- | --- | --- | --- | --- | --- | --- | --- | --- | --- | --- | --- | --- | --- | --- | --- | --- | --- | --- | --- | --- | --- | --- | --- | --- | --- | --- | --- | --- | --- | --- | --- | --- | --- | --- | --- | --- | --- | --- | --- | --- | --- | --- | --- | --- | --- | --- | --- | --- | --- | --- | --- | --- | --- | --- | --- | --- | --- | --- | --- | --- | --- | --- | --- | --- | --- | --- | --- | --- | --- | --- | --- | --- | --- | --- | --- | --- | --- | --- | --- | --- | --- | --- | --- | --- | --- | --- | --- | --- | --- | --- | --- | --- | --- | --- | --- | --- | --- | --- | --- | --- | --- | --- | --- | --- | --- | --- | --- | --- | --- | --- | --- | --- | --- | --- | --- | --- | --- | --- | --- | --- | --- | --- | --- | --- | --- | --- | --- | --- | --- | --- | --- | --- | --- | --- | --- | --- | --- | --- | --- | --- | --- | --- | --- | --- | --- | --- | --- | --- | --- | --- | --- | --- | --- | --- | --- | --- | --- | --- | --- | --- | --- | --- | --- | --- | --- | --- | --- | --- | --- | --- | --- | --- | --- | --- | --- | --- | --- | --- | --- |

Table S10. Separate control group vs treatment-concentration group pairwise ANOSIM results. * denotes a significant value of p ≤ 0.05. C1= control group 1, C2 = control group 2. L = low dose, H = high dose.

| **Phylum** |  |  |  |
| --- | --- | --- | --- |
| **Group 1** | **Group 2** | **R statistic** | **p-value** |
| C1 | C2 | 0.925 | 0.008* |
| C1 | Metal-H | 0.698 | 0.029* |
| C1 | Metal-L | 0.574 | 0.029* |
| C1 | Pesticide-H | 0.729 | 0.029* |
| C1 | Pesticide-L | 0.863 | 0.008* |
| C1 | Pharmaceutical-H | 0.926 | 0.029* |
| C1 | Pharmaceutical-L | 0.944 | 0.008* |
| C2 | Metal-H | 0.888 | 0.008* |
| C2 | Metal-L | 0.415 | 0.054 |
| C2 | Pesticide-H | 0.913 | 0.008* |
| C2 | Pesticide-L | 0.58 | 0.008* |
| C2 | Pharmaceutical-H | 0.538 | 0.018* |
| C2 | Pharmaceutical-L | 0.996 | 0.008* |
| **Family** |  |  |  |
| **Group 1** | **Group 2** | **R statistic** | **p-value** |
| C1 | C2 | 0.856 | 0.008* |
| C1 | Metal-H | 0.604 | 0.029* |
| C1 | Metal-L | 0.556 | 0.057 |
| C1 | Pesticide-H | 0.656 | 0.057 |
| C1 | Pesticide-L | 0.9 | 0.008* |
| C1 | Pharmaceutical-H | 0.815 | 0.029* |
| C1 | Pharmaceutical-L | 0.856 | 0.008* |
| C2 | Metal-H | 0.825 | 0.008* |
| C2 | Metal-L | 0.292 | 0.071 |
| C2 | Pesticide-H | 0.963 | 0.008* |
| C2 | Pesticide-L | 0.6 | 0.008* |
| C2 | Pharmaceutical-H | 0.59 | 0.018* |
| C2 | Pharmaceutical-L | 0.984 | 0.008* |
| **Species** |  |  |  |
| **Group 1** | **Group 2** | **R statistic** | **p-value** |
| C1 | C2 | 0.769 | 0.008* |
| C1 | Metal-H | 0.552 | 0.029* |
| C1 | Metal-L | 0.481 | 0.086 |
| C1 | Pesticide-H | 0.625 | 0.057 |
| C1 | Pesticide-L | 0.856 | 0.008* |
| C1 | Pharmaceutical-H | 0.833 | 0.029* |
| C1 | Pharmaceutical-L | 0.725 | 0.008* |
| C2 | Metal-H | 0.781 | 0.008* |
| C2 | Metal-L | 0.262 | 0.143 |
| C2 | Pesticide-H | 0.956 | 0.008* |
| C2 | Pesticide-L | 0.672 | 0.008* |
| C2 | Pharmaceutical-H | 0.651 | 0.018* |
| C2 | Pharmaceutical-L | 0.916 | 0.008* |

Table S11. SIMPER analysis results comparing phyla between treatments. Average abundance (Av. Abun), contribution (Cont. %) and cumulative contribution (Cum. %) are shown for the top five contributing taxa.

| **Control - Metal (Average dissimilarity = 6.85)** | | | | |
| --- | --- | --- | --- | --- |
| **Taxa** | **Av. Abun (C)** | **Av. Abun (M)** | **Cont. %** | **Cum. %** |
| Bacteroidota | 0.37 | 0.33 | 7.48 | 7.48 |
| Cyanobacteria | 0.36 | 0.39 | 6.15 | 13.63 |
| Verrucomicrobiota | 0.33 | 0.3 | 5.43 | 19.07 |
| Actinobacteriota | 0.16 | 0.19 | 5.26 | 24.33 |
| Proteobacteria | 0.4 | 0.4 | 5.22 | 29.55 |
| **Control - Pesticide (Average dissimilarity = 6.88)** | | | | |
| **Taxa** | **Av. Abun (C)** | **Av. Abun (Pe)** | **Cont. %** | **Cum. %** |
| Cyanobacteria | 0.36 | 0.4 | 7.58 | 7.58 |
| Bacteroidota | 0.37 | 0.34 | 6.27 | 13.85 |
| Verrucomicrobiota | 0.33 | 0.3 | 5.49 | 19.34 |
| Proteobacteria | 0.4 | 0.4 | 4.81 | 24.15 |
| Actinobacteriota | 0.16 | 0.19 | 4.71 | 28.86 |
| **Control - Pharmaceutical (Average dissimilarity = 6.64)** | | | | |
| **Taxa** | **Av. Abun (C)** | **Av. Abun (Ph)** | **Cont. %** | **Cum. %** |
| Cyanobacteria | 0.36 | 0.39 | 6.77 | 6.77 |
| Bacteroidota | 0.37 | 0.34 | 5.91 | 12.68 |
| Verrucomicrobiota | 0.33 | 0.3 | 5.52 | 18.2 |
| Actinobacteriota | 0.16 | 0.19 | 5.45 | 23.65 |
| Proteobacteria | 0.4 | 0.4 | 4.97 | 28.62 |
| **Metal - Pesticide (Average dissimilarity = 5.57)** | | | | |
| **Taxa** | **Av. Abun (M)** | **Av. Abun (Pe)** | **Cont. %** | **Cum. %** |
| Cyanobacteria | 0.39 | 0.4 | 7.04 | 7.04 |
| Bacteroidota | 0.33 | 0.34 | 6.43 | 13.47 |
| Proteobacteria | 0.4 | 0.4 | 3.94 | 17.41 |
| Planctomycetota | 0.23 | 0.22 | 3.83 | 21.24 |
| Desulfobacterota_G_459544 | 0.23 | 0.23 | 3.77 | 25.01 |
| **Metal - Pharmaceutical (Average dissimilarity = 5.31)** | | | | |
| **Taxa** | **Av. Abun (M)** | **Av. Abun (Ph)** | **Cont. %** | **Cum. %** |
| Bacteroidota | 0.33 | 0.34 | 6.7 | 6.7 |
| Cyanobacteria | 0.39 | 0.39 | 6.3 | 13 |
| Proteobacteria | 0.4 | 0.4 | 4.21 | 17.21 |
| Planctomycetota | 0.23 | 0.22 | 3.79 | 21 |
| Patescibacteria | 0.18 | 0.18 | 3.69 | 24.69 |
| **Pesticide - Pharmaceutical (Average dissimilarity = 5.04)** | | | | |
| **Taxa** | **Av. Abun (Pe)** | **Av. Abun (Ph)** | **Cont. %** | **Cum. %** |
| Cyanobacteria | 0.4 | 0.39 | 7.91 | 7.91 |
| Bacteroidota | 0.34 | 0.34 | 5.09 | 13.01 |
| Patescibacteria | 0.18 | 0.18 | 4.15 | 17.16 |
| Desulfobacterota_G_459544 | 0.23 | 0.23 | 4.09 | 21.25 |
| Chloroflexota | 0.25 | 0.25 | 3.4 | 24.65 |

Table S12. SIMPER analysis results comparing families between treatments. Average abundance (Av. Abun), contribution (Cont. %) and cumulative contribution (Cum. %) are shown for the top ten contributing taxa. Taxa that could not be classified down to family are written as “Phylum; lowest possible classification; f__”. C = control, M = metal treatment, Ph = pharmaceutical treatment, Pe = pesticide treatment.

| **Control - Metal (Average dissimilarity = 14.24)** | | | | |
| --- | --- | --- | --- | --- |
| **Taxa** | **Av. Abun (C)** | **Av. Abun (M)** | **Cont. %** | **Cum. %** |
| Coleofasciculaceae | 0.34 | 0.36 | 0.92 | 0.92 |
| Nostocaceae | 0.08 | 0.08 | 0.8 | 1.72 |
| VadinHA17_877549 (Bacteroidota) | 0.24 | 0.22 | 0.75 | 2.48 |
| Porticoccaceae | 0.16 | 0.15 | 0.74 | 3.21 |
| Prolixibacteraceae | 0.13 | 0.11 | 0.68 | 3.89 |
| JAAYJT01 (Bacteroidota) | 0.1 | 0.09 | 0.58 | 4.47 |
| UBA9973 (Patescibacteria) | 0.14 | 0.12 | 0.57 | 5.04 |
| Parachlamydiaceae | 0.07 | 0.09 | 0.5 | 5.54 |
| UBA11358 (Verrucomicrobiota) | 0.12 | 0.1 | 0.48 | 6.02 |
| Lentimicrobiaceae | 0.1 | 0.09 | 0.48 | 6.49 |
| **Control - Pesticide (Average dissimilarity = 14.40)** | | | | |
| **Taxa** | **Av. Abun (C)** | **Av. Abun (Pe)** | **Cont. %** | **Cum. %** |
| Coleofasciculaceae | 0.34 | 0.39 | 1.28 | 1.28 |
| Nostocaceae | 0.08 | 0.03 | 1.1 | 2.37 |
| Porticoccaceae | 0.16 | 0.13 | 0.8 | 3.18 |
| VadinHA17_877549 (Bacteroidota) | 0.24 | 0.23 | 0.76 | 3.94 |
| UBA11358 (Verrucomicrobiota) | 0.14 | 0.12 | 0.59 | 4.53 |
| Prolixibacteraceae | 0.13 | 0.11 | 0.58 | 5.11 |
| JAAYJT01 (Bacteroidota) | 0.1 | 0.1 | 0.52 | 5.63 |
| Bin106 (Bdellovibrionota) | 0.1 | 0.11 | 0.48 | 6.11 |
| UBA8199 (Verrucomicrobiota) | 0.15 | 0.13 | 0.46 | 6.57 |
| Verrucomicrobiota; Pedosphaerales; f__ | 0.11 | 0.09 | 0.46 | 7.04 |
| **Control - Pharmaceutical (Average dissimilarity = 14.00)** | | | | |
| **Taxa** | **Av. Abun (C)** | **Av. Abun (Ph)** | **Cont. %** | **Cum. %** |
| Coleofasciculaceae | 0.34 | 0.37 | 1.05 | 1.05 |
| Nostocaceae | 0.08 | 0.06 | 0.89 | 1.93 |
| Porticoccaceae | 0.16 | 0.14 | 0.79 | 2.72 |
| VadinHA17_877549 (Bacteroidota) | 0.24 | 0.23 | 0.73 | 3.45 |
| Prolixibacteraceae | 0.13 | 0.11 | 0.6 | 4.05 |
| UBA9973 (Patescibacteria) | 0.14 | 0.12 | 0.6 | 4.65 |
| JAAYJT01 (Bacteroidota) | 0.1 | 0.1 | 0.51 | 5.17 |
| Bin106 (Bdellovibrionota) | 0.1 | 0.11 | 0.5 | 5.66 |
| Verrucomicrobiota; Pedosphaerales; f__ | 0.11 | 0.09 | 0.44 | 6.11 |
| Opitutaceae | 0.08 | 0.06 | 0.44 | 6.55 |
| **Metal - Pesticide (Average dissimilarity = 12.84)** | | | | |
| **Taxa** | **Av. Abun (M)** | **Av. Abun (Pe)** | **Cont. %** | **Cum. %** |
| Nostocaceae | 0.08 | 0.03 | 1.17 | 1.17 |
| Coleofasciculaceae | 0.36 | 0.39 | 1.03 | 2.2 |
| Porticoccaceae | 0.15 | 0.13 | 0.76 | 2.96 |
| VadinHA17_877549 (Bacteroidota) | 0.22 | 0.23 | 0.63 | 3.59 |
| Bin106 (Bdellovibrionota) | 0.09 | 0.11 | 0.56 | 4.15 |
| JAAYJT01 (Bacteroidota) | 0.09 | 0.1 | 0.51 | 4.66 |
| UBA9973 (Patescibacteria) | 0.12 | 0.12 | 0.51 | 5.17 |
| Prolixibacteraceae | 0.11 | 0.11 | 0.48 | 5.65 |
| Burkholderiaceae | 0.08 | 0.1 | 0.46 | 6.11 |
| Parachlamydiaceae | 0.09 | 0.09 | 0.45 | 6.56 |
| **Metal - Pharmaceutical (Average dissimilarity = 12.45)** | | | | |
| **Taxa** | **Av. Abun (M)** | **Av. Abun (Ph)** | **Cont. %** | **Cum. %** |
| Nostocaceae | 0.08 | 0.06 | 0.95 | 0.95 |
| Coleofasciculaceae | 0.36 | 0.37 | 0.87 | 1.82 |
| Porticoccaceae | 0.15 | 0.14 | 0.75 | 2.57 |
| VadinHA17_877549 (Bacteroidota) | 0.22 | 0.23 | 0.59 | 3.16 |
| Bin106 (Bdellovibrionota) | 0.09 | 0.11 | 0.57 | 3.73 |
| JAAYJT01 (Bacteroidota) | 0.09 | 0.1 | 0.51 | 4.24 |
| Prolixibacteraceae | 0.11 | 0.11 | 0.5 | 4.74 |
| UBA9973 (Patescibacteria) | 0.12 | 0.12 | 0.48 | 5.22 |
| Parachlamydiaceae | 0.09 | 0.09 | 0.47 | 5.69 |
| Xanthobacteraceae | 0.13 | 0.12 | 0.46 | 6.15 |
| **Pesticide - Pharmaceutical (Average dissimilarity = 11.94)** | | | | |
| **Taxa** | **Av. Abun (Pe)** | **Av. Abun (Ph)** | **Cont. %** | **Cum. %** |
| Coleofasciculaceae | 0.39 | 0.37 | 1.06 | 1.06 |
| Nostocaceae | 0.03 | 0.06 | 0.97 | 2.03 |
| VadinHA17_877549 (Bacteroidota) | 0.23 | 0.23 | 0.6 | 2.64 |
| Porticoccaceae | 0.13 | 0.14 | 0.55 | 3.19 |
| UBA9973 (Patescibacteria) | 0.12 | 0.12 | 0.54 | 3.73 |
| Bin106 (Bdellovibrionota) | 0.11 | 0.11 | 0.4 | 4.13 |
| Parachlamydiaceae | 0.09 | 0.09 | 0.37 | 4.51 |
| DSM-16500 (Proteobacteria) | 0.06 | 0.06 | 0.37 | 4.87 |
| Anaerolineaceae | 0.14 | 0.14 | 0.36 | 5.23 |
| Gemmataceae | 0.09 | 0.09 | 0.36 | 5.59 |

Table S13. SIMPER analysis results comparing species between treatments. Average abundance (Av. Abun), contribution (Cont. %) and cumulative contribution (Cum. %) are shown for the top 15 contributing taxa. Taxa that could not be classified down to species are written as “Phylum; lowest possible classification; s__”. C = control, M = metal treatment, Ph = pharmaceutical treatment, Pe = pesticide treatment.

| **Control - Metal (Average dissimilarity = 19.20)** | | | | |
| --- | --- | --- | --- | --- |
| **Taxa** | **Av. Abun (C)** | **Av. Abun (M)** | **Cont. %** | **Cum. %** |
| SIO2C1 sp010672925 (Cyanobacteria) | 0.34 | 0.36 | 0.48 | 0.48 |
| Bacteroidota; VadinHA17_877549; s__ | 0.23 | 0.22 | 0.39 | 0.87 |
| Proteobacteria; SZUA-272; s__ | 0.14 | 0.14 | 0.35 | 1.22 |
| Cyanobacteria; Dolichospermum; s__ | 0.06 | 0.06 | 0.35 | 1.57 |
| JAAFHB01 sp010906965 (Bacteroidota) | 0.11 | 0.09 | 0.34 | 1.91 |
| UBA9973 sp000989515 (Patescibacteria) | 0.08 | 0.06 | 0.31 | 2.22 |
| Bacteroidota; JAAYJT01; s__ | 0.1 | 0.09 | 0.29 | 2.51 |
| UBA11358 sp003455565 (Verrucomicrobiota) | 0.1 | 0.08 | 0.25 | 2.76 |
| Verrucomicrobiota; Pedosphaerales; s__ | 0.11 | 0.09 | 0.24 | 3 |
| Proteobacteria; HB2-32-21; s__ | 0.08 | 0.07 | 0.24 | 3.24 |
| Cyanobacteria; Kamptonema; s__ | 0.03 | 0.04 | 0.23 | 3.47 |
| Actinobacteriota; PALSA-647; s__ | 0.06 | 0.08 | 0.23 | 3.69 |
| Nemorincola caseinilytica | 0.04 | 0.02 | 0.22 | 3.92 |
| Lentimicrobium saccharophilum | 0.09 | 0.09 | 0.22 | 4.14 |
| Verrucomicrobiota; Pedosphaerales; s__ | 0.09 | 0.07 | 0.21 | 4.35 |
| **Control - Pesticide (Average dissimilarity = 19.40)** | | | | |
| **Taxa** | **Av. Abun (C)** | **Av. Abun (Pe)** | **Cont. %** | **Cum. %** |
| SIO2C1 sp010672925 (Cyanobacteria) | 0.34 | 0.39 | 0.67 | 0.67 |
| Cyanobacteria; Dolichospermum; s__ | 0.06 | 0.02 | 0.44 | 1.11 |
| Bacteroidota; VadinHA17_877549; s__ | 0.23 | 0.22 | 0.39 | 1.5 |
| Proteobacteria; SZUA-272; s__ | 0.14 | 0.12 | 0.34 | 1.84 |
| Proteobacteria; HB2-32-21; s__ | 0.08 | 0.05 | 0.33 | 2.17 |
| JAAFHB01 sp010906965 (Bacteroidota) | 0.11 | 0.1 | 0.28 | 2.45 |
| Cyanobacteria; Nostocaceae; s__ | 0.04 | 0.01 | 0.28 | 2.73 |
| Bacteroidota; JAAYJT01; s__ | 0.1 | 0.09 | 0.26 | 2.99 |
| Patescibacteria; UBA9973; s__ | 0.11 | 0.09 | 0.25 | 3.24 |
| Bin106 sp002238945 (Bdellovibrionota) | 0.1 | 0.11 | 0.25 | 3.49 |
| UBA9973 sp000989515 (Patescibacteria) | 0.08 | 0.07 | 0.24 | 3.74 |
| Verrucomicrobiota; Pedosphaerales; s__ | 0.11 | 0.09 | 0.24 | 3.98 |
| Anabaena sp001858025 | 0.03 | 0.01 | 0.24 | 4.22 |
| Cyanobacteria; Kamptonema; s__ | 0.03 | 0.01 | 0.22 | 4.43 |
| ELB16-189 sp001567185 (Bacteroidota) | 0.04 | 0.02 | 0.22 | 4.65 |
| **Control - Pharmaceutical (Average dissimilarity = 19.04)** | | | | |
| **Taxa** | **Av. Abun (C)** | **Av. Abun (Ph)** | **Cont. %** | **Cum. %** |
| SIO2C1 sp010672925 (Cyanobacteria) | 0.34 | 0.37 | 0.54 | 0.54 |
| Bacteroidota; VadinHA17_877549; s__ | 0.23 | 0.22 | 0.37 | 0.92 |
| Cyanobacteria; Dolichospermum; s__ | 0.06 | 0.05 | 0.34 | 1.26 |
| Proteobacteria; SZUA-272; s__ | 0.14 | 0.12 | 0.33 | 1.59 |
| Proteobacteria; HB2-32-21; s__ | 0.08 | 0.06 | 0.32 | 1.91 |
| JAAFHB01 sp010906965 (Bacteroidota) | 0.11 | 0.1 | 0.29 | 2.2 |
| Bin106 sp002238945 (Bdellovibrionota) | 0.1 | 0.11 | 0.26 | 2.46 |
| Bacteroidota; JAAYJT01; s__ | 0.1 | 0.09 | 0.26 | 2.71 |
| Patescibacteria; UBA9973; s__ | 0.11 | 0.1 | 0.25 | 2.96 |
| Cyanobacteria; Nostocaceae; s__ | 0.04 | 0.03 | 0.24 | 3.19 |
| UBA9973 sp000989515 (Patescibacteria) | 0.08 | 0.07 | 0.23 | 3.43 |
| Verrucomicrobiota; Pedosphaerales; s__ | 0.11 | 0.09 | 0.23 | 3.66 |
| Proteobacteria; Aquicella; s__ | 0.04 | 0.05 | 0.21 | 3.87 |
| Anammoximicrobium | 0.07 | 0.05 | 0.21 | 4.08 |
| VBCG01 (Proteobacteria) | 0.07 | 0.08 | 0.21 | 4.28 |
| **Metal - Pesticide (Average dissimilarity = 17.84)** | | | | |
|  | **Av. Abun (M)** | **Av. Abun (Pe)** | **Cont. %** | **Cum. %** |
| SIO2C1 sp010672925 (Cyanobacteria) | 0.36 | 0.39 | 0.53 | 0.53 |
| Cyanobacteria; Dolichospermum; s__ | 0.06 | 0.02 | 0.5 | 1.03 |
| Cyanobacteria; Kamptonema; s__ | 0.04 | 0.01 | 0.41 | 1.44 |
| Proteobacteria; SZUA-272; s__ | 0.14 | 0.12 | 0.35 | 1.79 |
| Bacteroidota; VadinHA17_877549; s__ | 0.22 | 0.22 | 0.32 | 2.1 |
| UBA9973 sp000989515 (Patescibacteria) | 0.06 | 0.07 | 0.3 | 2.4 |
| Bin106 sp002238945 (Bdellovibrionota) | 0.09 | 0.11 | 0.29 | 2.69 |
| Cyanobacteria; Nostocaceae; s__ | 0.03 | 0.01 | 0.24 | 2.93 |
| JAAFHB01 sp010906965 (Bacteroidota) | 0.09 | 0.1 | 0.24 | 3.17 |
| Bacteroidota; JAAYJT01; s__ | 0.09 | 0.09 | 0.23 | 3.4 |
| Patescibacteria; UBA9973; s__ | 0.11 | 0.09 | 0.22 | 3.62 |
| Proteobacteria; Burkholderiaceae; s__ | 0.07 | 0.09 | 0.21 | 3.84 |
| Lentimicrobium saccharophilum | 0.09 | 0.09 | 0.21 | 4.05 |
| Proteobacteria; HB2-32-21; s__ | 0.07 | 0.05 | 0.2 | 4.25 |
| Cyanobacteria; Dolichospermum; s__ | 0.03 | 0.01 | 0.2 | 4.45 |
| **Metal - Pharmaceutical (Average dissimilarity = 17.50)** | | | | |
| **Taxa** | **Av. Abun (M)** | **Av. Abun (Ph)** | **Cont. %** | **Cum. %** |
| SIO2C1 sp010672925 (Cyanobacteria) | 0.36 | 0.37 | 0.44 | 0.44 |
| Cyanobacteria; Dolichospermum; s__ | 0.06 | 0.05 | 0.41 | 0.85 |
| Proteobacteria; SZUA-272; s__ | 0.14 | 0.12 | 0.34 | 1.19 |
| Bacteroidota; VadinHA17_877549; s__ | 0.22 | 0.22 | 0.29 | 1.48 |
| Bin106 sp002238945 (Bdellovibrionota) | 0.09 | 0.11 | 0.29 | 1.77 |
| JAAFHB01 sp010906965 (Bacteroidota) | 0.09 | 0.1 | 0.24 | 2.01 |
| Bacteroidota; JAAYJT01; s__ | 0.09 | 0.09 | 0.23 | 2.24 |
| Cyanobacteria; Kamptonema; s__ | 0.04 | 0.03 | 0.23 | 2.47 |
| Patescibacteria; UBA9973; s__ | 0.11 | 0.1 | 0.22 | 2.7 |
| UBA9973 sp000989515 (Patescibacteria) | 0.06 | 0.07 | 0.21 | 2.91 |
| Lentimicrobium saccharophilum | 0.09 | 0.09 | 0.21 | 3.11 |
| Proteobacteria; HB2-32-21; s__ | 0.07 | 0.06 | 0.2 | 3.31 |
| Cyanobacteria; Nostocaceae; s__ | 0.03 | 0.03 | 0.2 | 3.51 |
| Proteobacteria; Burkholderiaceae; s__ | 0.07 | 0.08 | 0.18 | 3.69 |
| Patescibacteria; UBA2206; s__ | 0.02 | 0.03 | 0.18 | 3.87 |
| **Pesticide - Pharmaceutical (Average dissimilarity = 16.96)** | | | | |
| **Taxa** | **Av. Abun (Pe)** | **Av. Abun (Ph)** | **Cont. %** | **Cum. %** |
| SIO2C1 sp010672925 (Cyanobacteria) | 0.39 | 0.37 | 0.53 | 0.53 |
| Cyanobacteria; Dolichospermum; s__ | 0.02 | 0.05 | 0.41 | 0.95 |
| Bacteroidota; VadinHA17_877549; s__ | 0.22 | 0.22 | 0.3 | 1.25 |
| Cyanobacteria; Kamptonema; s__ | 0.01 | 0.03 | 0.25 | 1.5 |
| UBA9973 sp000989515 (Patescibacteria) | 0.07 | 0.07 | 0.25 | 1.74 |
| Proteobacteria; SZUA-272; s__ | 0.12 | 0.12 | 0.24 | 1.98 |
| Patescibacteria; UBA9973; s__ | 0.09 | 0.1 | 0.23 | 2.2 |
| Bin106 sp002238945 (Bdellovibrionota) | 0.11 | 0.11 | 0.2 | 2.41 |
| Silvanigrella paludirubra | 0.01 | 0.02 | 0.2 | 2.61 |
| Anabaena sp001858025 | 0.01 | 0.02 | 0.19 | 2.8 |
| VBCG01 (Proteobacteria) | 0.08 | 0.08 | 0.18 | 2.98 |
| Acidobacteriota; Vicinamibacterales; s__ | 0.1 | 0.1 | 0.18 | 3.16 |
| Cyanobacteria; Dolichospermum; s__ | 0.01 | 0.02 | 0.18 | 3.34 |
| Cyanobacteria; Cyanobiaceae; s__ | 0.05 | 0.05 | 0.18 | 3.51 |
| Patescibacteria; UBA4665; s__ | 0.05 | 0.05 | 0.17 | 3.68 |

Table S14. Macroinvertebrate abundance table. C = control, M = metal treatment, Ph = pharmaceutical treatment, Pe = pesticide treatment. L = low dose, H = high dose.

| **Phylum** | Annelida | Arthropoda | | | | | | | | | | | | | | | | Mollusca |
| --- | --- | --- | --- | --- | --- | --- | --- | --- | --- | --- | --- | --- | --- | --- | --- | --- | --- | --- |
| **Class** | Clitellata | Arachnida | | | Collembola | Insecta | | | | | | | | | | | | Bivalvia |
| **Subclass** | Oligochaeta | Acari | | | ­ | Pterygota | | | | | | | | | | | | Autobranchia |
| **Order** | ­ | Trombidiformes | Oribatida | Mesostigmata | Symphypleona | Coleoptera | | | | | Diptera | | | | Hemiptera | | Lepidoptera | Sphaeriida |
| **Superfamily** | ­ | Trombidioidea | ­ | ­ | Sminthuroidea | Dytiscoidea | | | Sphaeriusoidea | Curculionoidea | Chironomoidea | | | Tipuloidea | Mesovelioidea | Gerroidea | Pyraloidea | Sphaerioidea |
| **Family** | ­ | ­ | ­ | ­ | Sminthurididae | Dytiscidae | | | Sphaeriusidae | Curculionidae | Chironomidae | | | Tipulidae | Mesoveliidae | Veliidae | Crambidae | Sphaeriidae |
| **Subfamily** | ­ | ­ | ­ | ­ | ­ | Lancetinae | Hydroporinae | Cybistrinae | ­ | Bagoinae | Podonominae | Chironominae | Tanypodinae | ­ | Mesovelia | Microvelia | ­ | ­ |
| **Genus** | ­ | ­ | ­ | ­ | Sminthurides | Lancetes | Sternopriscus | Cybister | Sphaerius | Bagous | ­ | ­ | ­ | ­ | ­ | ­ | ­ | ­ |
| **C-1** | 6 |  | 20 | 12 |  |  |  |  |  |  |  |  |  |  |  |  |  |  |
| **C-2** | 1 |  | 31 | 8 |  |  |  |  |  |  |  |  |  |  |  |  |  |  |
| **C-3** |  |  | 8 | 4 |  | 1 |  |  |  |  |  | 1 |  | 1 |  |  |  |  |
| **C-4** |  |  | 11 | 6 |  |  | 1 |  |  | 1 |  |  |  | 1 |  |  |  |  |
| **C-5** |  |  | 16 | 4 |  |  |  |  |  |  |  |  |  | 1 |  |  |  |  |
| **C-6** |  |  | 8 | 3 |  |  |  |  |  |  | 2 | 60 | 23 | 1 |  |  |  |  |
| **C-7** |  |  | 7 | 2 |  |  | 1 |  |  |  |  |  |  | 2 |  |  |  |  |
| **C-8** |  |  | 11 | 5 | 2 |  | 1 |  |  |  |  |  |  |  |  |  |  |  |
| **C-9** |  |  | 28 | 15 |  | 1 |  |  |  |  |  | 7 |  |  |  |  |  |  |
| **C-10** |  |  | 14 | 14 |  |  |  |  |  |  |  |  |  |  |  |  |  |  |
| **C-11** |  | 1 | 17 | 12 |  | 1 |  |  |  |  |  |  |  |  |  |  |  |  |
| **C-12** |  |  | 46 | 11 |  |  |  |  |  |  |  |  |  |  |  |  |  |  |
| **C-13** |  |  | 11 | 6 |  |  |  | 1 |  |  |  |  |  |  |  |  |  |  |
| **C-14** |  |  | 38 | 20 |  |  | 1 |  |  |  |  |  |  |  |  |  |  |  |
| **C-15** |  |  | 78 | 34 |  |  | 1 | 1 |  |  |  |  |  |  |  | 1 |  |  |
| **M-L-1** |  |  | 12 | 6 |  | 1 |  |  |  |  |  | 6 |  |  |  | 1 | 1 |  |
| **M-L-2** |  |  | 8 | 4 |  | 1 |  |  |  |  |  | 4 |  |  |  | 1 |  |  |
| **M-L-3** |  |  | 31 | 13 |  |  |  |  |  |  |  | 2 |  |  |  |  |  |  |
| **M-L-4** |  |  | 7 | 2 | 1 |  |  |  |  |  |  | 2 |  |  |  |  |  |  |
| **M-L-5** | 1 |  | 15 | 8 |  |  | 1 |  |  |  |  | 4 |  |  |  |  |  |  |
| **M-H-1** |  |  | 43 | 28 |  |  |  |  |  |  |  | 2 |  | 1 |  |  |  |  |
| **M-H-2** |  |  | 30 | 8 |  |  |  |  |  |  | 1 | 18 | 1 |  |  |  |  |  |
| **M-H-3** |  |  | 3 | 2 |  |  |  |  |  |  |  | 1 |  |  |  |  |  |  |
| **M-H-4** | 1 |  | 13 | 7 |  |  |  |  |  |  |  | 1 |  |  |  | 1 |  | 1 |
| **M-H-5** |  |  | 1 |  |  |  |  |  |  | 2 |  | 1 |  |  |  |  | 1 |  |
| **Pe-L-1** |  |  | 5 | 2 |  |  |  |  |  |  |  |  |  |  |  |  |  |  |
| **Pe-L-2** |  |  | 29 | 12 |  |  |  | 1 |  |  |  |  |  |  |  |  |  |  |
| **Pe-L-3** |  |  | 50 | 21 | 1 | 1 |  |  |  |  |  | 5 |  |  |  |  |  |  |
| **Pe-L-4** | 1 |  | 43 | 5 |  |  |  |  |  |  |  |  |  |  | 1 |  |  |  |
| **Pe-L-5** | 1 |  | 35 | 18 |  |  |  |  |  |  |  |  |  |  |  | 1 |  |  |
| **Pe-H-1** |  |  | 36 | 15 |  |  |  |  |  |  |  | 1 |  |  |  |  |  |  |
| **Pe-H-2** | 2 |  | 4 | 2 |  |  | 1 |  |  |  |  | 1 |  |  |  |  |  |  |
| **Pe-H-3** |  |  | 40 | 9 |  |  | 1 |  |  |  | 1 | 79 | 41 |  |  |  |  |  |
| **Pe-H-4** |  |  | 13 | 6 |  |  |  |  |  | 1 | 5 | 136 | 29 |  |  |  |  |  |
| **Pe-H-5** |  |  | 9 | 4 |  |  |  |  |  |  |  | 12 |  |  |  |  |  |  |
| **Ph-L-1** |  |  | 11 | 5 |  | 1 |  |  |  |  |  | 1 |  |  |  |  |  |  |
| **Ph-L-2** |  |  | 19 | 10 |  |  |  |  |  |  |  |  |  |  |  |  |  |  |
| **Ph-L-3** |  |  | 3 |  |  |  | 1 |  |  |  |  |  |  |  |  |  |  |  |
| **Ph-L-4** |  |  | 18 | 8 |  |  | 1 |  |  |  |  | 1 |  |  |  |  |  |  |
| **Ph-L-5** |  |  | 17 | 2 |  | 1 |  |  |  |  |  |  |  |  |  |  |  |  |
| **Ph-H-1** |  |  | 20 | 5 |  |  |  |  | 1 |  |  | 5 |  |  |  |  |  |  |
| **Ph-H-2** |  |  | 17 | 9 |  |  | 1 |  |  |  |  | 3 |  |  |  |  |  |  |
| **Ph-H-3** | 1 |  | 6 | 2 |  |  |  |  |  |  |  |  |  |  |  |  |  |  |
| **Ph-H-4** |  |  | 22 | 2 |  |  | 1 |  |  |  |  | 1 |  |  |  |  |  |  |
| **Ph-H-5** |  |  | 17 | 9 |  | 1 | 1 |  |  |  |  | 2 |  |  |  |  |  |  |

Table S15. Daily temperatures and rainfall over the duration of microcosm deployment, and averages for those months (BOM 2025). OCT = October, NOV = November.

| **Month** | **Date** | **Day** | **Temps** | | **Rain** |
| --- | --- | --- | --- | --- | --- |
|  |  |  | **Min** | **Max** |  |
|  |  |  | **°C** | **°C** | **mm** |
| OCT | 17 | Th | 15.4 | 25.3 | 0 |
|  | 18 | Fr | 17.7 | 22.4 | 2.4 |
|  | 19 | Sa | 12.3 | 18 | 36.4 |
|  | 20 | Su | 11.9 | 19.4 | 0.6 |
|  | 21 | Mo | 9 | 27.4 | 0 |
|  | 22 | Tu | 13.2 | 29.5 | 0 |
|  | 23 | We | 11.1 | 21.2 | 0 |
|  | 24 | Th | 6.7 | 18.5 | 0 |
|  | 25 | Fr | 7.7 | 15.4 | 0 |
|  | 26 | Sa | 1.7 | 23.1 | 0 |
|  | 27 | Su | 11.4 | 27.7 | 0 |
|  | 28 | Mo | 9.8 | 17.6 | 0 |
|  | 29 | Tu | 7.4 | 21 | 0 |
|  | 30 | We | 10.3 | 22.4 | 0 |
|  | 31 | Th | 10.5 | 19.7 | 0 |
| NOV | 1 | Fr | 10 | 16.9 | 0 |
|  | 2 | Sa | 4.2 | 26.2 | 0 |
|  | 3 | Su | 13.1 | 23.6 | 0 |
|  | 4 | Mo | 9.3 | 19.8 | 0 |
|  | 5 | Tu | 6.5 | 26.8 | 0 |
|  | 6 | We | 14.7 | 34.5 | 0 |
|  | 7 | Th | 11.3 | 23.2 | 0 |
|  | 8 | Fr | 8 | 20 | 1.6 |
|  | 9 | Sa | 5.4 | 20.9 | 0.2 |
|  | 10 | Su | 6.9 | 21.1 | 0.2 |
|  | 11 | Mo | 7.8 | 25.4 | 0 |
|  | 12 | Tu | 11.3 | 30.4 | 0 |
| Monthly averages (1991-2020 data): | | | | | |
| OCT | ­- | -­ | 10.9 | 20.5 | 55.8 |
| NOV | ­- | ­- | 12.8 | 22.9 | 63.3 |

Table S16. Water quality measurement results from weeks 1 (deployment), 2, and 3 of the 4 week experiment. No data was obtained at week 4 (conclusion) due to malfunctions with the field equipment. C = control, M = metal treatment, Ph = pharmaceutical treatment, Pe = pesticide treatment. L = low dose, H = high dose.

| Week | Treatment | EC (mS/cm) | DO (%) | pH | Temp (˚C) | Turb (ntu) |
| --- | --- | --- | --- | --- | --- | --- |
| Week 1 | Control | 60.4 | 83.7 | 6.3 | 13.0 | 205.0 |
|  | M-L | 63.2 | 81.4 | 6.3 | 11.8 | 171.0 |
|  | M-H | 60.7 | 83.5 | 6.3 | 12.0 | 165.0 |
|  | Pe-L | 67.9 | 81.4 | 6.3 | 12.0 | 156.0 |
|  | Pe-H | 55.5 | 82.5 | 6.5 | 13.3 | 166.0 |
|  | Ph-L | 59.0 | 84.7 | 6.4 | 12.6 | 192.0 |
|  | Ph-H | 68.7 | 77.0 | 6.2 | 11.4 | 178.5 |
| Week 2 | Control | 71.9 | 81.7 | 5.9 | 14.0 | 94.0 |
|  | M-L | 67.6 | 91.1 | 6.2 | 15.6 | 74.4 |
|  | M-H | 70.0 | 80.5 | 6.0 | 14.3 | 98.3 |
|  | Pe-L | 74.8 | 74.5 | 6.0 | 13.5 | 89.5 |
|  | Pe-H | 63.8 | 83.1 | 6.4 | 15.9 | 73.1 |
|  | Ph-L | 63.6 | 83.0 | 6.2 | 15.6 | 77.0 |
|  | Ph-H | 67.0 | 86.6 | 6.1 | 14.9 | 105.0 |
| Week 3 | Control | 83.3 | 78.1 | 6.2 | 12.6 | 72.4 |
|  | M-L | 77.1 | 81.8 | 5.4 | 13.2 | 52.9 |
|  | M-H | 69.3 | 82.5 | 5.6 | 12.6 | 46.0 |
|  | Pe-L | 79.4 | 76.9 | 5.2 | 13.9 | 55.4 |
|  | Pe-H | 70.4 | 79.3 | 5.5 | 12.9 | 40.9 |
|  | Ph-L | 74.4 | 81.1 | 5.4 | 13.1 | 56.4 |
|  | Ph-H | 81.5 | 75.2 | 5.3 | 13.6 | 53.0 |
